# Q134R: Small chemical compound with NFAT inhibitory properties improves behavioral performance and synapse function in mouse models of amyloid pathology

**DOI:** 10.1111/acel.13416

**Published:** 2021-06-12

**Authors:** Pradoldej Sompol, Jenna L. Gollihue, Susan D. Kraner, Irina A. Artiushin, Ryan A. Cloyd, Emad A. Chishti, Shon A. Koren, Grant K. Nation, Jose F. Abisambra, Orsolya Huzian, Lajos I. Nagy, Miklos Santha, Laszlo Hackler, Laszlo G. Puskas, Christopher M. Norris

**Affiliations:** ^1^ Sanders‐Brown Center on Aging University of Kentucky College of Medicine Lexington KY USA; ^2^ Center for Translational Research in Neurodegenerative Disease University of Florida Gainesville FL USA; ^3^ Avidin Ltd Szeged Hungary; ^4^ ELKH Biological Research Centre Szeged Hungary; ^5^ Aperus Pharma Co Ltd Szeged Hungary

**Keywords:** alzheimer's disease, calcineurin, dementia, nuclear factor of activated T cells, small chemical compound, synapses

## Abstract

Inhibition of the protein phosphatase calcineurin (CN) ameliorates pathophysiologic and cognitive changes in aging rodents and mice with aging‐related Alzheimer's disease (AD)‐like pathology. However, concerns over adverse effects have slowed the transition of common CN‐inhibiting drugs to the clinic for the treatment of AD and AD‐related disorders. Targeting substrates of CN, like the nuclear factor of activated T cells (NFATs), has been suggested as an alternative, safer approach to CN inhibitors. However, small chemical inhibitors of NFATs have only rarely been described. Here, we investigate a newly developed neuroprotective hydroxyquinoline derivative (Q134R) that suppresses NFAT signaling, without inhibiting CN activity. Q134R partially inhibited NFAT activity in primary rat astrocytes, but did not prevent CN‐mediated dephosphorylation of a non‐NFAT target, either in vivo, or in vitro. Acute (≤1 week) oral delivery of Q134R to APP/PS1 (12 months old) or wild‐type mice (3–4 months old) infused with oligomeric Aβ peptides led to improved Y maze performance. Chronic (≥3 months) oral delivery of Q134R appeared to be safe, and, in fact, promoted survival in wild‐type (WT) mice when given for many months beyond middle age. Finally, chronic delivery of Q134R to APP/PS1 mice during the early stages of amyloid pathology (i.e., between 6 and 9 months) tended to reduce signs of glial reactivity, prevented the upregulation of astrocytic NFAT4, and ameliorated deficits in synaptic strength and plasticity, without noticeably altering parenchymal Aβ plaque pathology. The results suggest that Q134R is a promising drug for treating AD and aging‐related disorders.

## Graphical abstract

pathophysiology including synapse dysfunction. Small chemical compound, Q134R, inhibits the activity of the downstream calcineurin‐dependent transcription factor NFAT (but does not inhibit calcineurin) and reduces synapse deficits and other AD biomarkers in APP/PS1 mice.

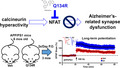

## 1 INTRODUCTION

Hyperactivation of the Ca^2+^/calmodulin‐dependent protein phosphatase, calcineurin (CN), is observed in aging and at early stages of Alzheimer's disease (AD) where it corresponds to the progression of multiple AD hallmarks including amyloid pathology, astrocyte reactivity, neurofibrillary tangles, and cognitive loss (Abdul et al., 2010; Reese & Taglialatela, 2011; Sompol & Norris, 2018). In experimental models of Aβ pathology, CN plays a causative or exacerbating role in several pathophysiologic processes like synapse loss/dysfunction, neuronal degeneration, glial reactivity and neuroinflammation, amyloid‐β (Aβ) peptide production, and impaired learning and memory (Dineley et al., 2007, 2010; Fernandez et al., 2016; Furman et al., 2012; Jin et al., 2012; Norris et al., 2005; Rojanathammanee et al., 2015; Dos Santos et al., 2020; Sompol et al., 2017; Taglialatela et al., 2009; Wu et al., 2010, 2012). In fact, many AD‐related biomarkers are reversed or ameliorated in experimental models by treatment with commercial CN‐inhibiting immunosuppressant drugs, such as tacrolimus (Dineley et al., 2007, 2010; Kuchibhotla et al., 2008; Stallings et al., 2018; Taglialatela et al., 2009). Moreover, an epidemiological study found that human kidney transplant patients treated with tacrolimus exhibit a significantly reduced risk for developing dementia compared with age‐matched subjects in the general population (Taglialatela et al., 2015). Together, these observations support the development of CN‐inhibiting strategies for treating AD and possibly other related neurodegenerative disorders.

A noted limitation of CN inhibitors (CNIs) for treating neurologic disorders is the risk of numerous and major adverse effects, which can include renal‐ and neuro‐dysfunction, as well as progressive lymphopenia (Farouk & Rein, 2020; Khalil et al., 2018). Although CN inhibitor toxicity is less likely to occur at “maintenance” doses commonly used in human solid organ transplant patients, the concerns raised suggest that alternative CN‐related targets may provide a safer approach for clinical applications beyond allograft transplant cases and autoimmune disorders. The nuclear factor of activated T cells (NFAT) is one of the best‐characterized and most widely studied CN substrates. NFATs reside in the cytosol when intracellular Ca^2+^ levels are low, but translocate to the nucleus upon binding and dephosphorylation by CN. In the nucleus, NFATs regulate the transcription of numerous genes and help trigger or shape striking phenotypic changes in target tissues (e.g., T‐cell activation, muscle fiber development and switching, vascular remodeling, and others) (Crabtree & Olson, 2002; de Frutos et al., 2007; Horsley & Pavlath, 2002; McCullagh et al., 2004; Sompol & Norris, 2018). NFATs are hyperactivated in the early stages of AD (Abdul et al., 2009) and are upregulated in amyloidogenic mouse models in parallel with CN alterations (Wu et al., 2010), especially in reactive astrocytes (Sompol et al., 2017).

Peptide‐based strategies have been used extensively in laboratory settings to prevent CN/NFAT interactions, without inhibiting CN activity *per se*. The most frequently used peptide for this purpose, VIVIT (Aramburu et al., 1999), improves many AD‐related biomarkers in experimental models of Aβ pathology and/or neurodegeneration (Furman et al., 2012; Hudry et al., 2012; Rojanathammanee et al., 2015; Sompol et al., 2017), similar to what is observed for CN inhibitors. However, the translational potential of VIVIT will very likely be limited by many of the same issues that plague other peptide/protein‐based reagents (i.e., poor stability, susceptibility to oxidation/hydrolysis, short half‐life, poor oral availability, and/or poor blood‐brain barrier penetrance; Lee et al., 2019).

Although small chemical compounds can provide many pharmacokinetic advantages over peptide/protein‐based drugs, small chemical inhibitors of NFAT activity (i.e., independent of CN inhibition) have only rarely been described (Sieber & Baumgrass, 2009). Dipyridamoles, which exhibit anti‐blood clotting and anti‐cancer properties, have been shown to inhibit NFAT signaling in several different cell types, but not CN activity (Mulero et al., 2009). These drugs can exhibit anti‐inflammatory actions (Guo et al., 2010) and may impart direct neuroprotection under certain conditions (Blake, 2004; Farinelli et al., 1998), similar to CN inhibitors. However, dipyridamoles also have cytotoxic properties and can exacerbate excitotoxicity under injurious conditions (Lobner & Choi, 1994), as well. Another class of chemical compounds, INCAs, were identified based on their propensity to mimic the NFAT‐inhibitory properties of the VIVIT peptide (Roehrl et al., 2004). Unfortunately, three of the major INCAs investigated (INCAs 1,2, and 6) all exhibited cytotoxic properties when studied in vivo (Kang et al.,l., 2005). The pyrazolopyrimidine derivative, NCI3, was also shown to prevent NFAT activation (Sieber et al., 2007), but little information is available regarding the impact of this compound in intact animals. It remains unclear whether any of these NFAT modulating agents lead to other adverse effects commonly associated with CN inhibitor drugs.

Recently, a new hydroxyquinoline derivative, Q134R, was described that exhibits strong cytoprotective properties (Kanizsai et al., 2018) and interactions with the hypoxia‐inducible factor (HIF)1 system (Hackler et al., 2019). Q134R is blood–brain barrier permeant and can be safely administered to experimental subjects across a wide dosing range (e.g., the LD_50_ is >90mg/kg in mice). A Phase 1a clinical trial confirmed that Q134R is also safe for consumption by humans (EudraCT Number: 2016‐000368‐40). Interestingly, screening of more than 150 molecular pathways suggested CN/NFAT signaling as an additional potential molecular target for Q134R. Here, we show that Q134R partially inhibits NFAT activity in primary neural cultures, but does not inhibit CN phosphatase activity, *per se*. Acute oral delivery of Q134R to two different mouse models of Aβ pathology prevented deficits in Y maze tasks. Chronic oral delivery of Q134R promoted survival in WT mice and caused no signs of lymphopenia. Moreover, long‐term treatment of APP/PS1 mice led to reduced NFAT4 expression in astrocytes, reduced glial reactivity markers, and improved synaptic function and plasticity, without affecting Aβ load. The results are consistent with previous studies that have advanced CN/NFAT inhibition as an alternative AD treatment, and/or as a complimentary strategy to the reduction of key AD pathologic hallmarks (i.e., using amyloid‐lowering biologics). Moreover, the lack of effect on CN activity, together with few signs of toxicity, suggests that Q134R could produce similar neurologic benefits compared with CNIs, with fewer adverse effects.

## 2 RESULTS

### 2.1 Q134R inhibits NFAT activity in astrocytes but has little to no effect on CN activity

**FIGURE 1:**
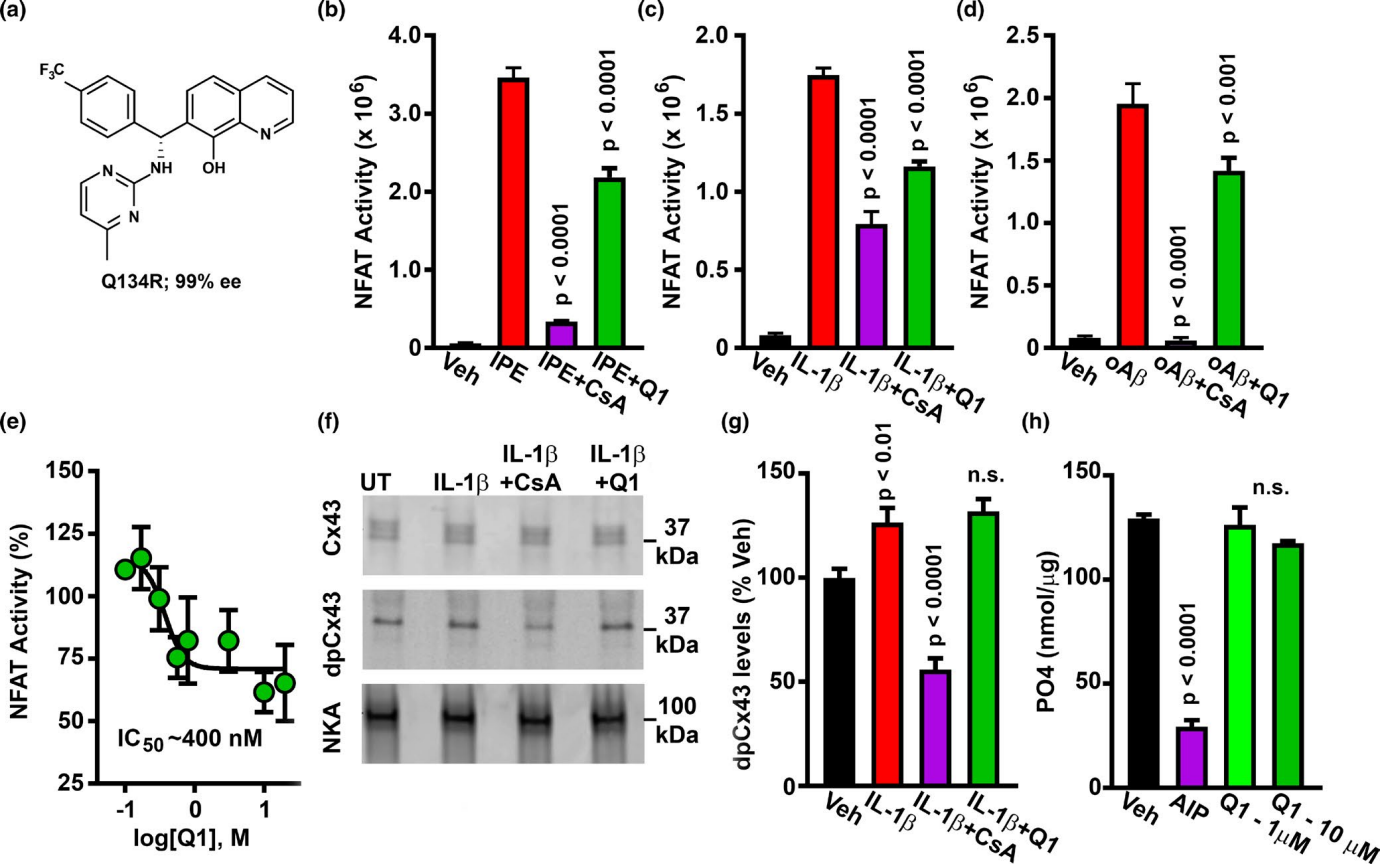
Q134R inhibits NFAT signaling but does not inhibit CN activity. (a) Chemical structure of Q134R. B‐D, Mean ± SEM NFAT‐dependent luciferase activity in primary astrocytes treated with the following CN/NFAT activators: 1 μM ionomycin and 1 μM phorbol ester (IPE) (b), 10 ng/ml IL‐1β (c), or 65 nM oligomeric Aβ peptides (d). CN/NFAT activators were also co‐delivered with the CN inhibitor, 5 μM CsA or 10 μM Q134R. Vehicle (Veh)‐treated cells served as a control. In each case, Q134R significantly inhibited NFAT‐luciferase expression. (e) Mean ± SEM NFAT‐luciferase expression (%) in primary astrocytes treated with 10 ng/ml IL‐1β in the presence of increasing Q134R concentrations. Q134R inhibited astrocytic NFAT signaling in a concentration‐dependent manner. (f) Representative western blot for total Cx43, dpCx43, and Na/K ATPAse loading control in primary astrocytes treated with Veh, or 10 ng/ml IL‐1β in the presence/absence of 5 μM CsA or 10 μM Q134R. Full blots are shown in Figure S2. (g) Mean ± SEM dpCx43 levels (% of Veh) under the conditions shown in panel f. CsA inhibited dephosphorylation of Cx43, but Q134R did not. (h) Mean ± SEM PO_4_ released from a synthetic phosphopeptide in response to in vitro CN activity. Dephosphorylation is inhibited by CN‐AIP (100 μM), but not by Q134R. For panels b‐e, *n* = six 35 mm dishes per condition. For panel h, *n* = 4 pooled samples per condition

Q134R is a cytoprotective and blood–brain barrier permeant hydroxyquinoline derivative (Figure 1a) developed by Avidin Ltd (Hackler et al., 2019; Kanizsai et al., 2018) and found safe for human consumption following a Phase 1A clinical trial (EudraCT Number: 2016‐000368‐40). Initial studies tested Q134R in the Cerep Diversity panel composed of binding and functional assays for ion channels, neurotransmitter receptors, enzymes (including 25 Zn‐ion containing enzymes), peptidases, and kinases along with other targets related to neuroprotection/neurodegeneration (data not shown). Results of the screening toward more than 150 cellular targets revealed NFATs as a potential candidate mechanism. Follow‐up studies in HEK 293 cells showed that NFAT‐controlled luciferase expression, induced by the addition of ionomycin (0.5 μM) and phorbol ester (IPE, 0.5 μM), to elevate intracellular Ca^2+^ levels and stimulate CN activity, was inhibited in a dose‐dependent manner by Q134R (IC_50_ of 566 nM; Figure S1a).

CN/NFAT affects neural function in disease models through multiple cell types including neurons (Hopp et al., 2018; Hudry et al., 2012; Ting et al., 2020; Wu et al., 2010), microglia (Ferrari et al., 1998; Kataoka et al., 2009; Nagamoto‐Combs & Combs, 2010; Mizuma et al., 2019), pericytes (Blanchard et al., 2020; Filosa et al., 2007), and astrocytes.

(Canellada et al., 2008; Fernandez et al., 2007; Furman et al., 2012; Norris et al., 2005; Perez‐Ortiz et al., 2008; Sama et al., 2008; Serrano‐Perez et al., 2011; Sompol et al., 2017). For our initial studies on Q134R, we chose to look at primary astrocytes because CN/NFAT changes in this cell type are particularly striking in intact animal models of aging, injury, and AD (Norris et al., 2005; Serrano‐Perez et al., 2011; Neria et al., 2013; Furman et al., 2016; Pleiss *et al*. 2016; Sompol et al., 2017) and also because the conditions for evoking CN/NFAT activity in primary astrocytes, by multiple disease‐relevant factors, have been well‐established and are highly reproducible (Abdul et al., 2009; Canellada et al., 2008; Fernandez et al., 2007; Furman et al., 2010; Sama et al., 2008).

Primary astrocytes were treated for 3 hr with ionomycin/phorbol ester (Figure 1b), IL‐1β (Figure 1c), or oligomeric Aβ (oAβ) peptides (Figure 1d) in the presence or absence of Q134R, or the CN inhibitor cyclosporine (CsA) (*n* = six 35 mm plates per condition). As expected, NFAT‐dependent luciferase expression in response to each activator was strongly inhibited by CsA (*p* < 0.0001). Q134R also significantly inhibited NFAT activity (*p* < 0.0001 under IPE and IL‐1β treatment and *p* < 0.001 under oAβ treatment), albeit to a lesser extent than CsA. Q134R‐mediated inhibition of astrocytic NFAT activity was dose dependent (IC_50_ ~400 nM) and partial (35–40% maximal inhibition) (Figure 1e). Using a saturating concentration of Q134R, we observed similar levels of inhibition of NFAT‐dependent luciferase activity in primary rat cortical neurons (Figure S1b).

**FIGURE 2:**
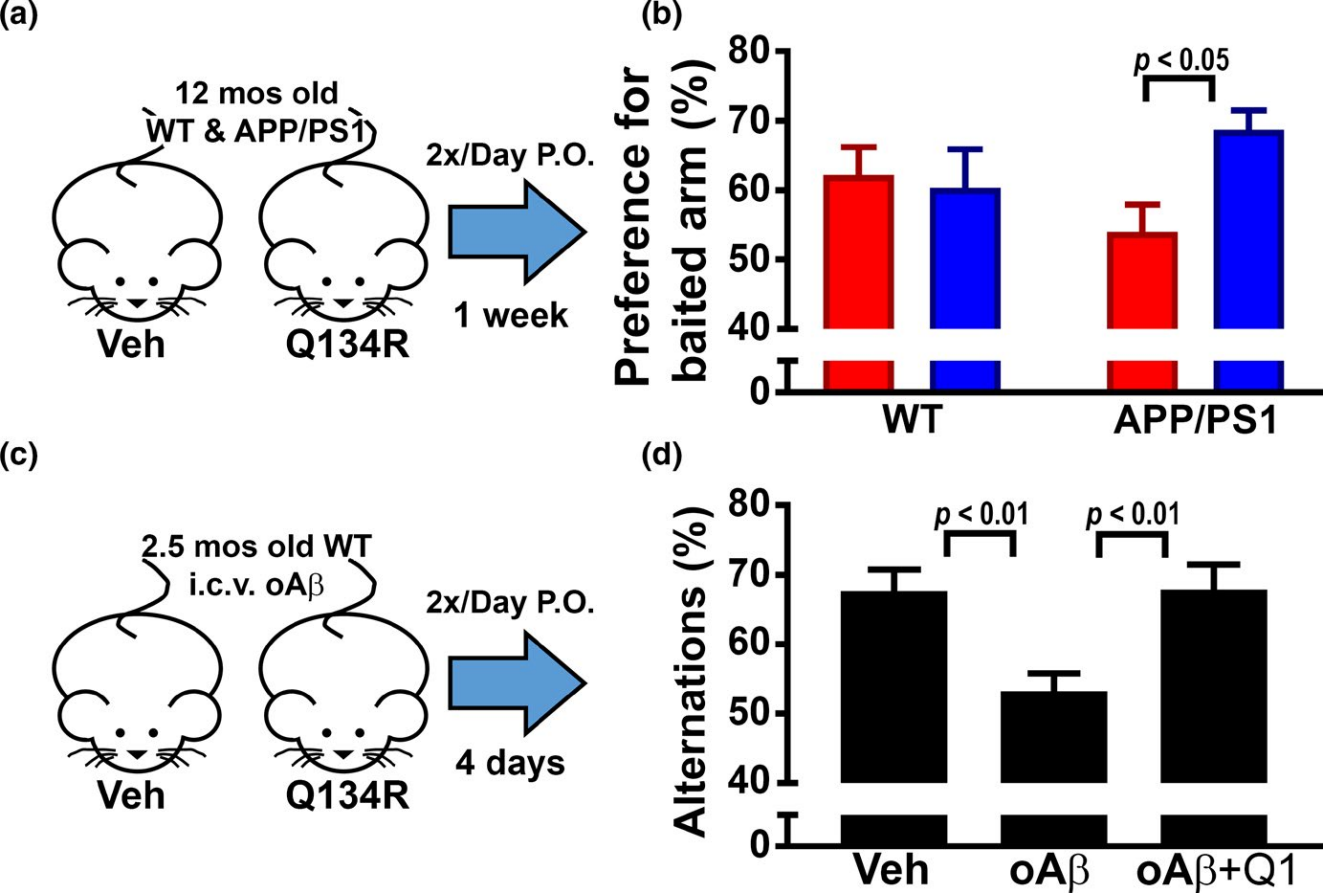
Acute treatment with Q134R improves cognitive function in mouse models of amyloid pathology. (a) 12‐month‐old WT and APP/PS1 littermates were oral‐gavaged 2×/day, for 7 days with vehicle (Veh) or Q134R (4 mg/kg) and then tested on a novel arm version of the Y maze. (b) Percent preference for “baited” arm (mean ± SEM) in WT and APP/PS1 mice. Q134R improved performance in APP/PS1 mice but did not alter the performance levels of WT mice. *n* = 9–10 mice per group. (c) 2.5‐month‐old WT mice received a single intracebroventricular injection of oAβ peptides (50 pmol total) or vehicle (Veh) and then were oral‐gavaged 2× daily with Veh or Q134R (4 mg/kg) for 4 days prior to the assessment of spontaneous alternations on the Y maze. (d) Mean ± SEM spontaneous alternations in mice treated with vehicle or oAβ in the presence or absence of Q134R. Q134R improved performance in oAβ‐treated mice. *n* = 7–8 mice per group

Most compounds found to inhibit NFAT signaling act on, or upstream of, CN. To assess whether Q134R is a CN inhibitor we investigated the CN‐dependent dephosphorylation of a non‐NFAT substrate in vivo and in vitro (Figure 1f‐h). For in vivo measures of CN activity (Figure 2f,g), we assessed the CN‐dependent dephosphorylation of connexin 43 (Cx43), which occurs in primary astrocytes in response to injurious stimuli (Li & Nagy, 2000; Tence et al., 2012) (Figure 1f, also Figure S2). Astrocytes were stimulated with IL‐1β in the presence or absence of the CN inhibitor CsA or Q134R. Cx43 levels were assessed using two different antibodies: one that labels total Cx43 (tCx43) and one that labels Cx43 only when it is dephosphorylated at Ser368 (dpCx43) (Li & Nagy, 2000; Tence et al., 2012). IL‐1β had little effect on total Cx43 levels but caused a 25–30% increase in dpCx43 levels (*p* < 0.01), an effect that was fully blocked by CsA (Figure 1g; *p* < 0.0001). In fact, dpCx43 levels in CsA‐treated cells were reduced to below the untreated condition, suggesting that basal levels of CN keep Cx43 in a relatively dephosphorylated state (at least at Ser368). In contrast, when used at the same concentration for maximally inhibiting NFAT activity (Figure 1e), Q134R had no effect on dpCx43 levels.

To assess CN activity in vitro, the amount of free phosphate released from a synthetic phosphopeptide substrate was quantified in the presence of human CN Aα/CN B heterodimer activated by Ca^2+^/calmodulin (Figure 1h). The addition of the CN autoinhibitory peptide (CN‐AIP 100 μM; a strong and highly specific inhibitor of CN phosphatase activity) suppressed free phosphate levels, as expected (*p* < 0.0001). In contrast, Q134R, used at either 1 or 10 μM concentrations, had no effect on phosphate release, demonstrating that Q134R does not directly inhibit CN activity. Together, these experiments suggest that Q134R suppresses NFAT signaling, without affecting CN activity, per se.

### 2.2 Acute treatment with Q134R improves Y maze performance in APP/PS1 mice and wild‐type mice treated with oligomeric Aβ peptides

Acute treatments with commercial CNIs, like tacrolimus, have been shown to improve cognition in transgenic mouse models that overexpress Aβ (Dineley et al., 2007; Taglialatela et al., 2009) or in mice injected intracerebroventricularly with oligomeric Aβ (oAβ) peptides (Dineley et al., 2010). To determine whether acute Q134R treatment has similar cognitive enhancing effects, 12‐month‐old male APP/PS1 mice and 3‐ to 4‐month‐old male WT mice injected with oAβ were administered Q134R or vehicle across 4–7 days and tested on a Y maze.

As shown in Figure 2a and b, APP/PS1 mice and WT littermates were oral‐gavaged twice per day for 7 days with vehicle or Q134R (4 mg/kg) and tested on a novel arm version of the Y maze. Preference for a novel “baited” arm was assessed (*n* = 9–10 mice per treatment group) and the results revealed a significant interaction between drug treatment and genotype [*F*(1,34) = 3.96, *p* = 0.05]. Vehicle‐treated APP/PS1 mice generally performed worse than their WT counterparts, though this difference was not significant. Moreover, Q134R significantly improved performance in APP/PS1 (*p* < 0.05), but not in WT mice (Figure 2b). For Aβ‐infusion studies, WT mice received a single ICV injection of oAβ_42_ peptides (50 pmol total) under anesthesia. Mice were then recovered and treated twice daily via oral gavage with Q134R (4 mg/kg) or vehicle for four additional days before testing on a spontaneous alternation version of the Y maze (Figure 2c,d; *n* = 7–8 mice per condition). As shown in Figure 2d, oAβ injections led to impaired alternation performance in the absence of Q134R treatment (*p* < 0.001). However, no cognitive deficit was observed in oAβ_42_ ‐injected mice treated with Q134R. Together, these observations suggest that acute Q134R treatment improves cognitive function in rodent models of AD‐like pathology.

### 2.3 Chronic delivery of Q134R does not alter immune cell profiles, but promotes long‐term survival

**FIGURE 3:**
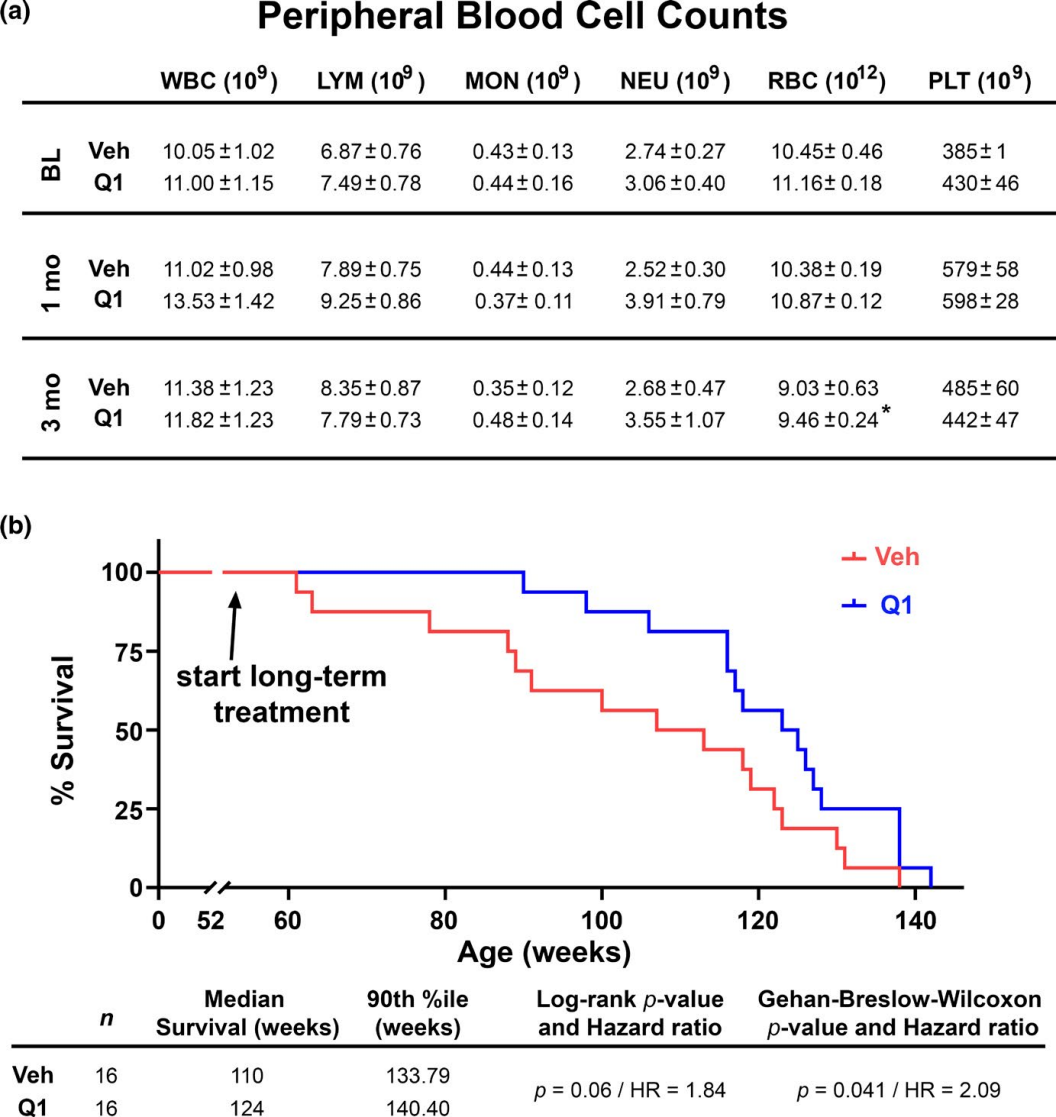
Chronic treatment with Q134R has little effect on white blood cell counts and enhances survival in adult mice. (a) Table showing mean ± SEM peripheral blood cell counts in 6‐month‐old WT mice measured before and at 4 and 12 weeks during treatment (2× daily, oral gavage) with vehicle (Veh) or Q134R (Q1, 4 mg/kg). Cell types include white blood cells (WBC), lymphocytes (LYM), monocytes (MON), neutrophils (NEU), red blood cells (RBC), and platelets. No effects of drug treatment or timepoint were found, except for RBCs, which showed a slight decrease in Q134R mice at 12 weeks relative to baseline. *n* = 9–10 mice per group; *p < 0.05, within group comparison of baseline vs. 12‐week time point. (b) Kaplan–Meier curves for WT mice gavaged with Veh or Q134R (Q1, 4 mg/kg). Drug treatment was initiated at 52 weeks of age and mice were followed until time of death. Q134R enhanced survival across the lifespan. *n* = 16 mice per group

Although CNIs often show benefits in rodent models of AD‐like pathology when given acutely (over a few days, or weeks), chronic treatment with these drugs (e.g., over months or years) causes systemic immunosuppression in otherwise healthy subjects, characterized, in part, by a drop in lymphocyte counts and other white blood cell changes (Gallo et al., 2006; Khalil et al., 2018; Koprak et al., 1996). Chronic lymphopenia could offset possible neurologic benefits achieved by long‐term CN inhibition. To determine if Q134R was associated with lymphopenia, we measured blood cell counts in male and female adult mice (~6 months of age) that were oral‐gavaged twice daily for 12 weeks with vehicle or Q134R at a dose associated with improved cognitive function (4 mg/kg, see Figure 2). Blood samples were collected at baseline (immediately prior to the first dosing session) and then again at 4 and 12 weeks of drug treatment for the quantification of lymphocyte (LYM), white blood cell (WBC), monocyte (MON), neutrophil (NEU), platelet (PLT), and red blood cell (RBC) counts (Figure 3a). With the exception of RBC counts, Q134R treatment had no significant effects on any of the cell types assessed. RBCs showed a small decrease across treatment (baseline vs. 12 weeks) for both vehicle and Q134R groups, with the reduction reaching significance (*p* < 0.05) in Q134R mice. No other overt signs of toxicity (e.g., weight loss) were observed. Although this study was not specifically powered to detect sex differences, the effects of Q134R were very similar across males and females. Thus, unlike CNIs, chronic treatment with Q134R does not appear to be associated with a reduction in peripheral immune cells, though there may be some impact on RBC levels.

To determine if chronic Q134R treatment negatively affects mortality, we treated middle‐age WT female mice over a nearly 2‐year‐long period. Starting at 52 weeks of age, mice were treated daily (via oral gavage, 4 mg/kg) with vehicle or Q134R (*n* = 16 mice/group) and followed until death. Kaplan–Meier curves are shown in Figure 3b and were used to derive median survival, which was greater for Q134R‐treated mice (124 vs. 110 weeks). In fact, Q134R treatment promoted survival across the duration of treatment (Log‐rank test, *p* = 0.06, Hazard ratio = 1.84; Gehan–Breslow–Wilcoxon test, *p* = 0.041, Hazard ratio = 2.09), though lifespan was not appreciably extended. Together, the results suggest that long‐term, chronic treatment with Q134R is safe.

### 2.4 Chronic treatment with Q134R tends to limit glial reactivity in APP/PS1 mice without affecting Aβ pathology

**FIGURE 4:**
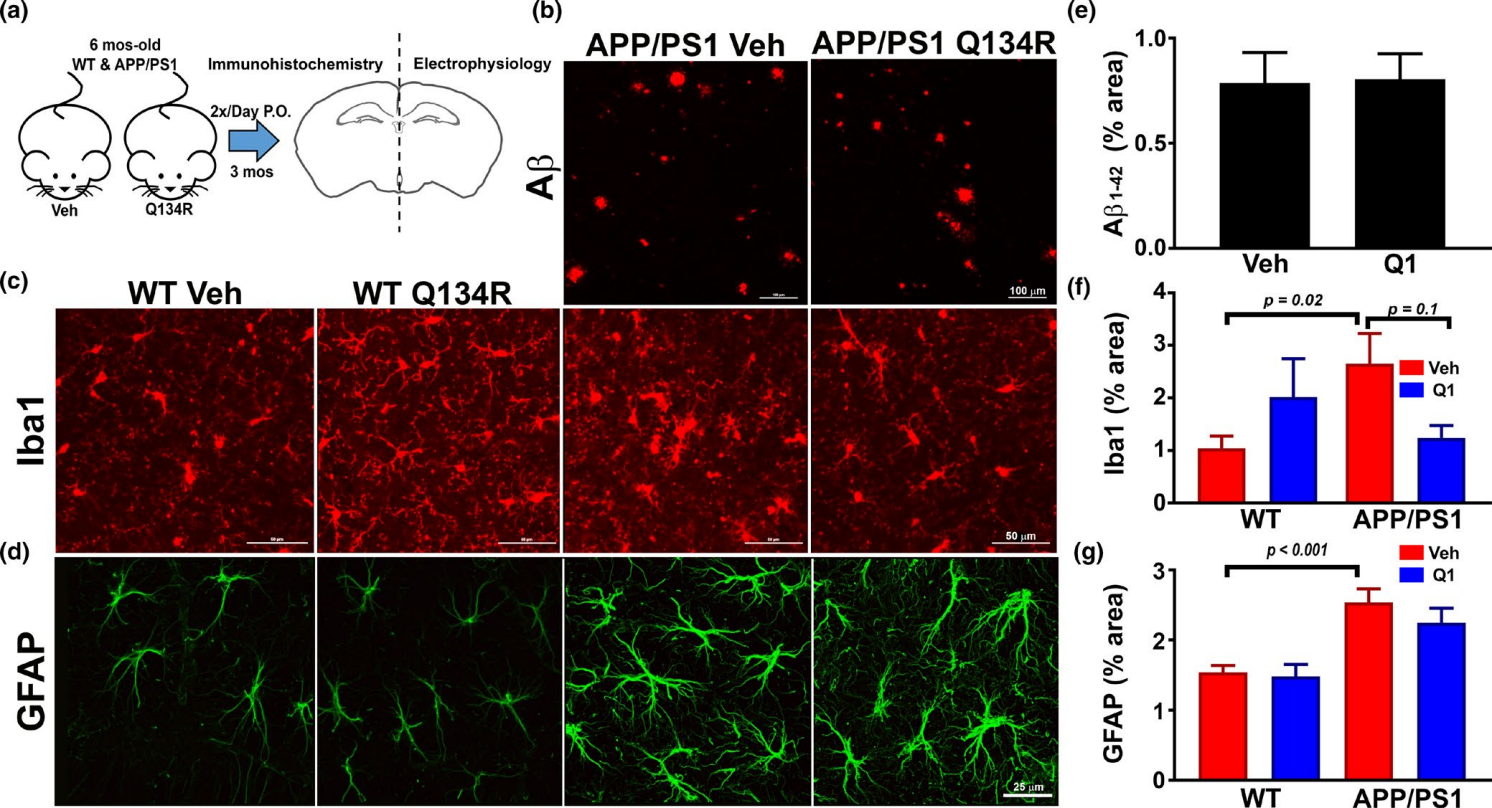
Chronic treatment with Q134R reduces tends to limit glial activation, but does not alter Aβ plaque load. (a) Illustration of experimental treatment conditions. Six‐month‐old APP/PS1 mice were oral gavaged 2×/day with vehicle (Veh) or Q134R (4 mg/kg) for 3 months. At the endpoint, one brain hemisphere was formalin‐fixed for immunolabeling, while the other hemisphere from some mice was used for the preparation of in situ brain slices for hippocampal electrophysiology (Figure 6). (b) Confocal micrographs showing labeling for Aβ_1‐42_ in neocortex of APP/PS1 mice treated with vehicle (Veh) or Q134R (Q1) (calibration bars = 100 μm). (c and d) Confocal micrographs showing co‐labeling for Iba1 (c, red) or GFAP (d, green) in the hippocampus of WT and APP/PS1 mice treated with Veh or Q1 (calibration bars = 50 and 25 μm). (e‐f), Mean ± SEM total labeling (% area) for Aβ_1‐42_ (e), Iba1 (f), and GFAP (g) in WT and APP/PS1 mice treated with Veh (red) or Q1 (blue). Iba1 and GFAP were both elevated in APP/PS1 mice, as expected. Q134R tended to reduce Iba1 levels on APP/PS1 mice, but did not significantly alter Aβ_1‐42_ plaque load or GFAP levels. *n* = 13–15 mice per genotype/drug group

We next investigated whether long‐term treatment with Q134R was associated with beneficial effects on several hallmark brain biomarkers of AD including elevated Aβ plaque load, and increased glial reactivity. Cohorts of male and female APP/PS1 mice and WT littermates were treated with Q134R (4 mg/kg, 2×/day P.O) or vehicle from 6 to 9 months of age. Coronal brain sections were then prepared and labeled with antibodies to Aβ (APP/PS1 mice only, *n* = 19 vehicle; *n* = 15 Q134R), Iba1 (marker for microglia, *n* = 10 WT vehicle; *n* = 8 WT Q134R; *n* = 10 APP/PS1 vehicle; *n* = 8 APP/PS1 Q134R), or GFAP (marker for reactive astrocytes *n* = 12 WT vehicle; *n* = 13 WT Q134R; *n* = 16 APP/PS1 vehicle; *n* = 17 APP/PS1 Q134R). Results are parsed into sex categories in Table 1. Overall results are shown in Figure 4.

**TABLE 1:** Immunolabeling measures: Drug × Sex effects within genotype groups

	WT Veh	WT Q1	APP/PS1 Veh	APP/PS1 Q1
Aβ				
M	‐‐	‐‐	0.38 ± 0.09	0.49 ± 0.14
F	‐‐	‐‐	0.92 ± 0.2^b^	0.97 ± 0.22
GFAP				
M	1.06 ± 0.16	0.92 ± 0.22	2.23 ± 0.28	1.60 ± 0.25
F	1.22 ± 0.29	1.73 ± 0.39	3.09 ± 0.40	3.14 ± 0.28^c^
Iba1				
M	1.43 ± 0.60	0.91 ± 0.35	2.22 ± 1.42	1.32 ± 0.42
F	1.26 ± 0.39	2.91 ± 1.16	2.74 ± 0.55	1.63 ± 0.58
NFAT4/GFAP				
M	0.53 ± 0.06	0.40 ± 0.04	0.90 ± 0.14	0.8 ± 0.14
F	0.58 ± 0.10	0.78 ± 0.16^a^	1.27 ± 0.17	0.94 ± 0.15
NFAT4/DAPI				
M	0.67 ± 0.13	0.74 ± 0.24	1.24 ± 0.26	1.01 ± 0.20
F	0.96 ± 0.34	0.97 ± 0.28	1.46 ± 0.23	1.31 ± 0.23

Note

All values are expressed as mean ± SEM labeling intensity (% area occupied). ns per group are indicated in text. Approximately 46% of mice were male.

^a^ vs. Male WT Q134R.

^b^ vs. Male APP/PS1 vehicle.

^c^ vs. Male APP/PS1 Q134R.

Although female APP/PS1 mice exhibited greater Aβ plaque loads relative to male mice (Table 1, *p* = 0.01), as previously reported (Jiao et al., 2016), no significant effect of Q134R was observed for either sex. Q134R also did not affect Aβ plaque load when males and females were combined (Figure 4b, e). For Iba1, we found a significant interaction between Q134R treatment and transgene [F(1,30) = 5.45, *p* = 0.03) (Figure 4c, f). APP/PS1 mice exhibited significantly greater Iba1 levels as expected (*p* = 0.02). Within the APP/PS1 group, Q134R tended to reduce Iba1 levels overall (*p* = 0.1), and no sex differences in the extent of reduction were observed (Table 1). In contrast, Q134R was associated with a nonsignificant increase in Iba‐1 (*p* = 0.18) in WT mice, mostly attributable to an elevation in females (Table 1). For GFAP, we found a significant transgene effect *F*(1, 48) = 23.67, indicative of greater GFAP levels in APP/PS1 mice (*p* < 0.001), as expected (Figure 4d, g). Q134R had no significant effects on GFAP in either transgene group. However, within APP/PS1 mice, we did find a significant effect of sex [F(1,29) = 11.4, *p* = 0.002], indicating that GFAP levels were higher in APP/PS1 females, regardless of drug treatment (see Table 1). Interestingly, Q134R was associated with a modest nonsignificant reduction in GFAP in male APP/PS1 mice, whereas female APP/PS1 mice showed a slight increase in GFAP levels with Q134R treatment (Table 1). The results suggest that Q134R tends to limit glial activation in APP/PS1 mice, without affecting Aβ levels.

### 2.5 Chronic treatment with Q134R reduces NFAT4 expression in astrocytes, but has little effect on NFAT4 co‐localization with astrocyte nuclei

**FIGURE 5:**
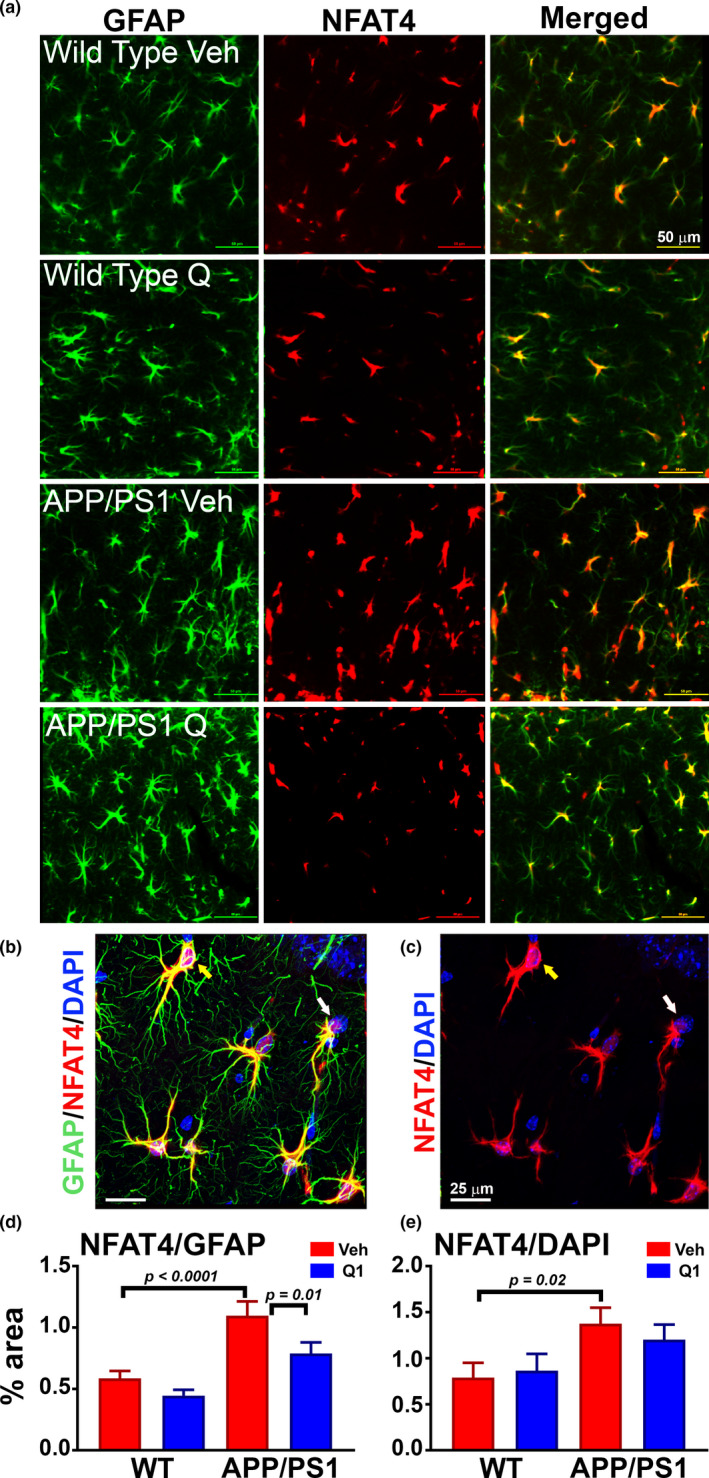
Chronic treatment with Q134R reduces total NFAT4 levels, but does not inhibit co‐localization with astrocyte nuclei. Six‐month‐old WT and APP/PS1 mice were oral gavaged 2×/day with vehicle (Veh) or Q134R (4 mg/kg) for 3 months as described in Figure 4. (a) Confocal micrographs showing the co‐localization of NFAT4 (red) with GFAP‐positive astrocytes (green) in the hippocampus of WT and APP/PS1 mice treated with vehicle (Veh) or Q134R (Q1) (calibration bars = 50 μm). (b‐c) High magnification Z stack images of GFAP, NFAT4, and DAPI. For clarity, NFAT4 and DAPI labels in the same field are shown without the green GFAP label in panel (c) Yellow arrows point to astrocyte nuclei that also express NFAT4 (pink/purple). White arrows point to astrocyte nuclei that are largely devoid of NFAT4 (blue). (d‐e) Mean ± SEM NFAT4 labeling presented as the percent area also occupied by GFAP (d) or occupied by DAPI (e). NFAT4 labeling intensity was increased in APP/PS1 vehicle‐treated mice, as expected. Q134R reduced total NFAT4 levels (co‐localized with GFAP), but did not significantly alter NFAT4 localization with DAPI‐labeled astrocyte nuclei. *n* = 13–15 mice per genotype/drug group

Several NFAT isoforms have been implicated in AD pathophysiology, two of which (NFAT3 and NFAT4) were investigated here. The NFAT3 isoform is strongly associated with neurons where it is thought to mediate the degeneration of dendrites/neurons, as well as altered expression of synapse‐related proteins (Shioda *et al*. 2006; Wu et al., 2010; Hopp et al., 2018). The NFAT4 isoform is selectively expressed in astrocytes, relative to neurons, and is generally increased in proportion to the expression of the astrocyte‐specific cytoskeletal protein GFAP during acute brain injury or chronic amyloid pathology (Furman et al., 2016; Neria et al., 2013; Serrano‐Perez et al., 2011; Sompol et al., 2017). Both NFAT isoforms showed strong labeling in WT and APP/PS1 mice within their respective cell types. However, neither transgene nor drug treatment had a discernable effect on NFAT3, which was almost totally confined to the dendritic regions of neurons and showed very little co‐localization with neuronal nuclei (Figure S3). In contrast, NFAT4 labeling (Figure 5a, 5d) was significantly affected by both transgene [F(1,51) = 27.43, *p* < 0.0001] and drug treatment [F(1,51) = 5.71, *p* = 0.02]. Astrocytes in APP/PS1 mice showed significantly greater NFAT4 labeling relative to WT mice (*p* < 0.0001, Figure 5d) and Q134R caused a significant reduction in total NFAT4 levels within the APP/PS1 group (*p* = 0.01, Figure 5d). In a previous study on 5xFAD mice (which show profound astrocyte reactivity and amyloid pathology), NFAT4 exhibited elevated co‐localization with astrocyte nuclei. Moreover, delivery of the NFAT‐inhibiting peptide, VIVIT, directly to astrocytes reduced NFAT nuclear localization in the 5xFAD model (Sompol et al., 2017). As shown in Figure 5b, 5c, and 5e, NFAT4 localization to astrocyte nuclei was elevated in APP/PS1 mice (Figure 5e *p* = 0.02), but was relatively insensitive to Q134R treatment. There were no effects of sex on NFAT labeling regardless of transgene (Table 1). These results show that Q134R modulates select NFAT properties in intact mouse models of amyloid pathology, though the mechanism of action remains unclear.

### 2.6 Chronic treatment with Q134R improves synaptic function in APP/PS1 mice

**FIGURE 6:**
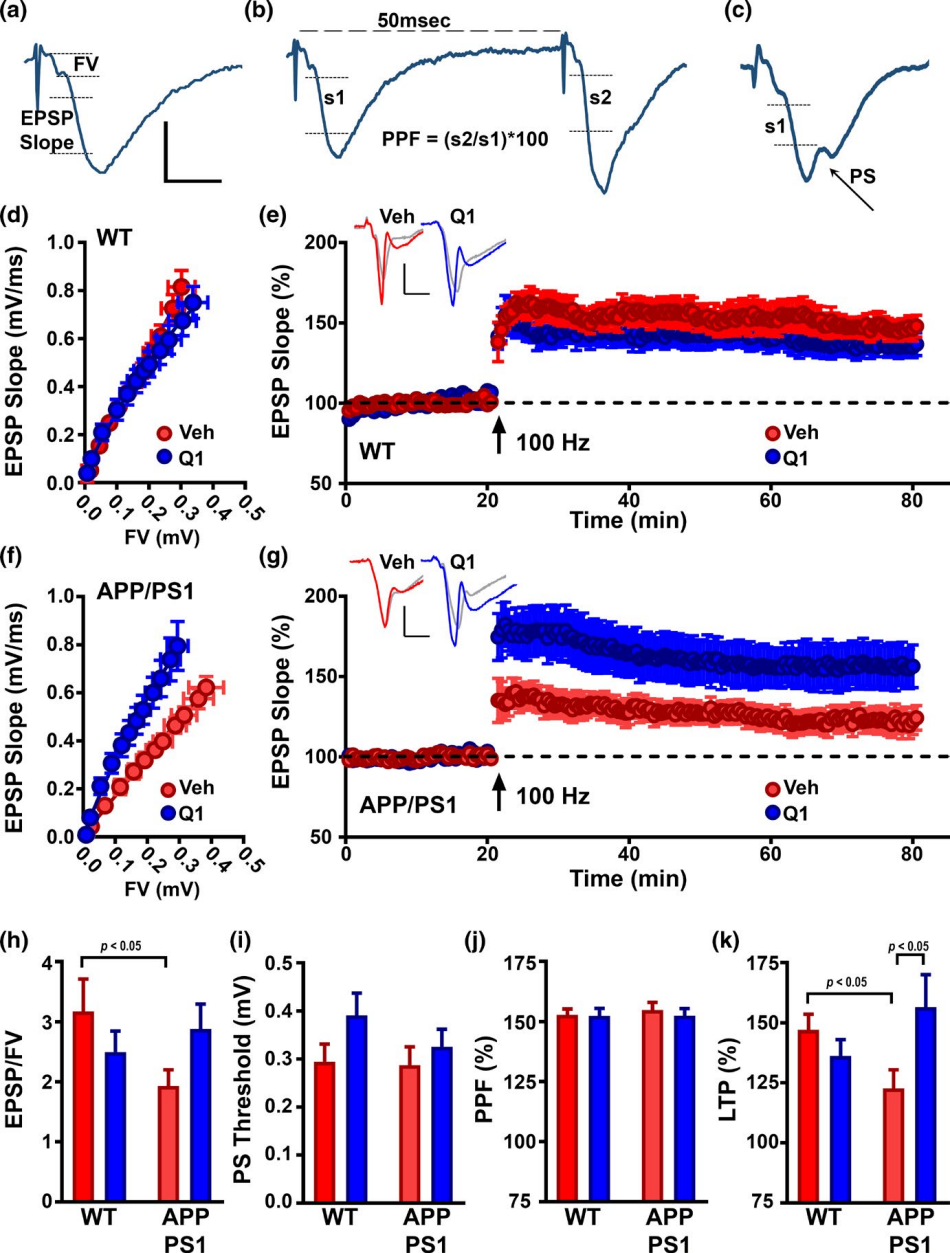
Chronic Q134R treatment improves hippocampal synaptic function and plasticity in APP/PS1 mice. Six‐month‐old WT and APP/PS1 mice were oral gavaged 2×/day with vehicle (Veh) or Q134R (4 mg/kg) for 3 months as described in Figures 4,5. In situ brain slices were prepared for electrophysiological analyses in hippocampal CA1 stratum radiatum. (a‐c) representative waveforms illustrating the synaptic parameters measured. EPSP slope to FV ratio (a) was measured to derive synaptic strength. Calibration bars: vertical = 0.5 mV, horizontal =5 ms. Twin stimulus pulses delivered 50 ms apart were used to elicit two successive field EPSPs (b). PPF was obtained by dividing the EPSP slope of pulse 2 (s2) by the EPSP slope of pulse 1 (s1) and multiplying by 100. The EPSP slope value measured at the first appearance of a PS in the ascending arm of the EPSP was used to assess PS Threshold (c). D‐G, Synaptic strength curves and LTP time plots for WT mice (d, e) and APP/PS1 mice (f, g), respectively. For synaptic strength curves (d, f), mean ± SEM EPSP slope amplitudes (vertical error bars) are plotted against corresponding mean ± SEM presynaptic FV amplitudes (horizontal error bars). For LTP plots (e, g), mean ± SEM EPSP slope values (% of baseline) are plotted during the 20 min prior to (baseline) and 60 min after the delivery of two 100 Hz (1‐s duration) stimulus trains (separated by 10 s). Calibration bars: vertical = 0.5 mV, horizontal = 5 ms. Treatment with Q134R shifted the synaptic strength curve to the left and enhanced LTP levels in APP/PS1 mice (f, g), but had little effect in WT mice. (h‐k) Mean ±SEM values for the EPSP/FV ratio (h), PS threshold (i), PPF (j), and LTP (k) at 60 min post 100 Hz stimulation. Q134R significantly increased the EPSP/FV ratio and enhanced LTP levels in APP/PS1 mice, but had little effect in WT mice. *n* = 8–14 mice per genotype/drug group

Electrophysiologic indices of synaptic transmission are exquisitely sensitive to brain injury and pathology and are frequently used to assess drug effects on brain function. Deficits in the most commonly investigated synaptic measures, basal synaptic strength and/or long‐term potentiation (LTP), are usually ameliorated by inhibition of CN or NFATs, via genetic or pharmacologic means (Chen et al., 2002; Dineley et al., 2010; Furman et al., 2012, 2016; Sompol et al., 2017). To determine if Q134R is associated with similar synaptoprotective properties, we collected electrophysiologic recordings from CA1 *stratum radiatum* in acutely prepared coronal brain slices from 9‐month‐old male and female APP/PS1 mice and WT littermates treated for 3 months with Q134R (4 mg/kg, 2×/day P.O) or vehicle as described in Figures 4, 5. Field potentials in CA1 were elicited in response to prodromal electrical stimulation of Schaffer collateral/commissural fibers (Figure 6a‐c). Several synaptic parameters including basal synaptic strength (synaptic strength curves shown in Figure 6d and 6f; maximum EPSP/FV ratio shown in Figure 6h), LTP (time plots shown in Figure 6e and 6g, mean LTP levels shown in Figure 6k), population spike (PS) threshold (Figure 6i), and paired pulse facilitation (PPF) (Figure 6j) were measured and compared across treatment groups (*n* = 8–14 mice, per treatment group).

As expected, both synaptic strength (*p* < 0.05) and LTP (*p* < 0.05) were reduced in vehicle‐treated APP/PS1 mice relative to their WT counterparts. Q134R ameliorated each of these effects: that is, treatment of APP/PS1 mice with Q134R shifted the synaptic strength curve to the left (Figure 6f), increased the EPSP‐to‐FV ratio (Figure 6h), and enhanced LTP levels (Figure 6g and 6k). In contrast, Q134R caused a small, albeit insignificant, reduction in synaptic strength and LTP in WT mice. No transgene or drug effects for PS threshold (a measure of neuronal excitability) and PPF (a measure of short‐term plasticity) were observed (Figure 6i and 6j, respectively). Electrophysiological results are further parsed for males and females in Table 2. The most notable effects of sex were observed in WT mice for maximum EPSP/FV ratio and PS threshold, both of which tended to be reduced in females. In contrast, deficits in LTP found in APP/PS1 mice tended to be greater in males and showed greater recovery with Q134R treatment. The results demonstrate that, like CN/NFAT inhibitors, Q134R protects synaptic function in mouse models of AD‐like pathology.

**TABLE 2:** Electrophysiology measures: Drug × Sex effects within genotype groups

	WT Veh	WT Q1	APP/PS1 Veh	APP/PS1 Q1
Max EPSP/FV				
M	4.10 ± 0.93	3.08 ± 0.66	2.38 ± 0.49	2.60 ± 0.57
F	2.25 ± 0.58^a^	2.1 ± 0.35	1.61 ± 0.27	3.07 ± 0.46^b^
PPF				
M	161 ± 11	159 ± 12	161 ± 12	148 ± 10
F	155 ± 12	169 ± 6	150 ± 5	174 ± 10
PS Threshold				
M	0.45 ± 0.10	0.37 ± 0.09	0.31 ± 0.07	0.27 ± 0.06
F	0.23 ± 0.03^a^	0.41 ± 0.05	0.27 ± 0.04	0.37 ± 0.05
LTP				
M	154 ± 8	152 ± 10	112 ± 12	167 ± 26c
F	141 ± 11	128 ± 8	132 ± 6	155 ± 14

Note

All values are expressed as mean ± SEM. ns per group are indicated in text. Approximately 44% of mice were male.

^a^ vs. Male WT vehicle.

^b^ vs. Male WT Q134R.

^c^ vs. Male APP/PS1 vehicle.

## 3 DISCUSSION

This study conducted both in primary cell cultures and in intact mice demonstrated that the novel hydroxyquinoline compound Q134R exhibits NFAT inhibitory properties in the absence of CN modulation. In an intact mouse model of AD‐like amyloid pathology, Q134R reduced the expression of the NFAT4 isoform, which is typically upregulated in reactive astrocytes. Similar to peptide‐based inhibitors of NFAT (i.e., VIVIT), Q134R improved cognitive and synaptic function, and generally promoted survival beyond mid‐age. Recently, Q134R was proven safe and well‐tolerated in a Phase 1A clinical trial (EudraCT Number: 2016‐000368‐40). Together with the present findings, these results suggest that Q134R is a promising drug candidate to treat and/or prevent dementia in patients with AD or AD‐related disorders.

### 3.1 Q134R and NFAT signaling

Q134R is a novel 8‐hydroxyquinoline derivative recently shown to exhibit cytoprotective effects along with HIF‐1A stabilization properties (Hackler et al., 2019). Further preliminary analyses of Q134R activity against >150 cellular targets revealed CN/NFAT signaling as a possible “hit.” This study is the first to characterize the NFAT inhibitory properties of Q134R in primary cells and intact animal models of AD‐like pathology, and among the very few studies to describe the actions of a small chemical NFAT inhibitor (Sieber & Baumgrass, 2009). As a first step in evaluating Q134R’s NFAT inhibitory properties, we chose to investigate astrocytes because NFATs (particularly NFAT4) exhibit marked upregulation and activation in astrocytes with injury and amyloid pathology (Furman et al., 2016; Serrano‐Perez et al., 2011; Sompol et al., 2017) and show reliable activation patterns in primary cultures (Fernandez et al., 2007) Sama et al., 2008; Abdul et al., 2009; Furman et al., 2010). In primary astrocytes, Q134R inhibited NFAT transcriptional regulation in response to exogenous Ca^2+^ mobilizing agents, and to pathogenic extracellular factors. Q134R‐mediated inhibition of NFAT activity was dose‐dependent, but only partial in extent. As a potential therapeutic strategy for managing neurodegenerative disorders, it is notable that Q134R had no apparent direct effects on CN activity *in vitro* or *in vivo*, suggesting that Q134R acts downstream of CN activation. These observations suggest that Q134R could be used to target key CN‐mediated deleterious processes without triggering many of the adverse effects commonly ascribed to commercial CN inhibitors (Farouk & Rein, 2020; Khalil et al., 2018) (see additional discussion below).

### 3.2 Effects of Q134R on intact mice

During the preclinical development of Q134R, 2‐week repeated dose toxicity studies (with 2‐week follow‐up periods) were conducted in the Wistar rat and the Beagle (data not shown). Overall, Q134R was devoid of major effects on the cardiovascular and central nervous systems. The determined “No Observed Adverse Effect Level” (NOAEL) and maximal tolerated dose (MTD) values were 30 mg/kg in the rat (NOAEL) and 12 mg/kg in the dog (MTD). When delivered at lower levels (4mg/kg, 2×/day) across a comparable time period (1 week), this study revealed that Q134R boosted cognition in mid‐aged APP/PS1 mice and wild‐type mice receiving intracerebral ventricular injection of oAβ peptides. The cognitive enhancing effects of Q134R were very similar to that reported for amyloid mouse models treated with commercial CNIs (Dineley et al., 2007, 2010; Taglialatela et al., 2009) suggesting a common mechanism of action between Q134R and CNIs.

A major downside to CNIs for the treatment of progressive neurodegenerative diseases is the systemic immunosuppression and toxicity that develops with chronic use. However, in contrast to CNIs, long‐term delivery of Q134R in mice elicited few obvious adverse effects. Indeed, Q134R was well tolerated when given orally over many weeks and did not lead to weight loss or progressive lymphopenia, which can happen with immunosuppressive CNIs (Gallo et al., 2006; Khalil et al., 2018; Koprak et al., 1996). Of the peripheral cell types that were assessed during treatment, only red blood cells showed a significant change over time (~16% reduction with Q134R over 3 months). Although the absolute number of red blood cells was still within the normal range for adult mice (Nemzek et al., 2001), this reduction could reflect a trend toward an anemic condition that is generally not associated with tacrolimus or other CN inhibitors. If this reduction is confirmed in future studies, additional investigation will be required to identify clinical symptoms of anemia and determine if the cause is related to impaired production and/or increased breakdown of red blood cells. Still, WT mice gavaged daily with Q134R survived for many months, and while absolute lifespan did not differ between placebo and Q134R‐treated mice, survival probability for the Q134R group was elevated at nearly every time point evaluated (for up to 88 weeks of treatment). These results suggest that Q134R is not only well‐tolerated, but it may have benefits beyond CNS function, highlighting the need to assess the cellular effects of Q134R on NFATs in other organ systems.

Oral administration of Q134R over 3 months reduced signs of glial reactivity, especially in microglia. Significant reductions in Iba‐1 have been similarly reported in APP/PS1 mice treated with the NFAT‐inhibitory peptide VIVIT (Furman et al., 2012l Rojanathammanee et al., 2015; Manocha et al., 2017). However, in spite of reducing astroglial NFAT4 expression, Q134R had only marginal effects on GFAP expression (particularly in females), suggesting that Q134R has stronger effects on microglia, and/or modulates very specific aspects of the reactive astrocyte phenotype that do not necessarily involve GFAP. This result is in contrast to previous results of VIVIT, or NFAT knock‐down, in amyloid mouse models (Furman et al., 2012; Manocha et al., 2017; Sompol et al., 2017), but is comparable to our previous work in a rat traumatic brain injury model, where astrocyte‐mediated delivery of VIVIT produced several beneficial effects on neural function, without appreciably modifying GFAP levels (Furman et al., 2016).

Chronic Q134R treatment also improved hippocampal synaptic function and plasticity in APP/PS1 mice. In humans, synapse dysfunction/loss is a very likely proximal cause of cognitive decline in the early stages of AD and AD‐related disorders (Scheff et al., 2015). Consequently, many, if not most, promising compounds for treating the clinical symptoms of AD are prescreened in relevant animal models using electrophysiologic measures of hippocampal synaptic transmission. Here, we found that chronic Q134R treatment improved both CA1 synaptic strength and LTP in APP/PS1 mice, suggesting that the cognitive‐boosting effects of this compound may follow directly from its beneficial actions on synapses. Similar to effects on glial markers, the synaptoprotective actions of Q134R mirror the actions of commercial CNIs and peptide‐based NFAT inhibitors reported in multiple animal models (Chen et al., 2002; Dineley et al., 2010; Furman et al., 2012, 2016; Sompol et al., 2017), again suggesting that hyperactive NFAT signaling is a primary target for Q134R. Of course, future studies will be needed to determine which aspects of synaptic transmission are specifically affected by Q134R (e.g., dendritic spine stability; glutamate receptor shuttling; impaired glutamate uptake, etc.). While synaptic parameters in WT mice were relatively less sensitive to Q134R, we did observe a mild (insignificant) drug‐related deficit in synaptic strength, indicating that there could be some potential for interfering with functional synaptic connections in healthy animals. And though these studies were not specifically powered to detect sex differences, Q134R tended to affect synaptic parameters differently in males and females (e.g., synaptic strength and LTP, Table 2). The mechanistic basis for this possible sexual dimorphism is not clear, but should be investigated more extensively in future studies.

Finally, it is notable that despite its other putative beneficial effects, Q134R did not significantly alter Aβ plaque load in double transgenic APP/PS1 mice. This may be a little surprising because BACE1 (the rate‐limiting enzyme for Aβ production) is transcriptionally induced in astrocytes by NFAT4 (Jin et al., 2012). Moreover, systemic delivery of VIVIT peptides and viral‐mediated delivery of VIVIT to astrocytes were shown to reduce Aβ pathology in both the double transgenic APP/PS1 mouse model (Furman et al., 2012; Rojanathammanee et al., 2015) and the more aggressive 5xFAD strain (Sompol et al., 2017). Why Q134R lacks amyloid‐lowering properties remains unclear but could relate to the overall weaker NFAT inhibitory properties of Q134R relative to VIVIT, which is a strong NFAT inhibitor (Aramburu et al., 1999). Q134R may also modulate other NFAT‐independent substrates that negate the amyloid‐lowering effects inherent to NFAT inhibition (see below for further discussion). Regardless, our findings are consistent with other studies showing that the reduction of Aβ, at least in preclinical models, is not a prerequisite for improving neural function and cognition (Joyashiki et al., 2011; Middeldorp et al., 2016; Tong et al., 2012; Voorhees et al., 2018).

### 3.3 Cellular mechanism of Q134R

A key unresolved issue of the present work centers on the precise mechanism for NFAT inhibition by Q134R. Unlike the NFAT inhibitory peptide VIVIT, Q134R did not appear to alter the subcellular localization of NFATs in either astrocytes (Figure 5e) or neurons (Figure S3b), though Q134R did reduce astrocytic NFAT4 labeling intensity. It is possible that the relatively mild pathology associated with 9‐month‐old APP/PS1 mice, combined with the milder NFAT‐inhibitory properties of Q134R, is not ideal for obtaining easy‐to‐measure differences in NFAT nuclear shuttling intact animals. Alternatively, Q134R may inhibit NFATs through a novel mechanism. Possibilities include: disruption of NFAT interactions with DNA binding elements; impaired interactions with other transcription factors; activation of NFAT kinases; and/or suppression of NFAT expression (as suggested in Figure 5d). Pinning down the exact mechanism of Q134R‐NFAT interactions was beyond the scope of this study, but investigations are underway to test each of these possibilities.

Experiments are also underway to determine if Q134R similarly affects different NFAT isoforms expressed across multiple neural cell types. Initial studies found that saturating levels of Q134R result in similar levels of NFAT inhibition in neurons (Figure S1), where hyperactivation of the NFAT pathway has been implicated in AD‐related neurite degeneration and synapse dysregulation (Hopp et al., 2018; Hudry et al., 2012; Wu et al., 2010, 2012). Thus, the protective actions of Q134R in intact animals are probably not limited to astrocytes. The CN/NFAT pathway in microglia has also been shown to mediate/modulate pathophysiologic effects relevant to AD including neuroinflammation (Nagamoto‐Combs & Combs, 2010; Rojanathammanee et al., 2015; Manocha et al., 2017). Here, we found that Q134R reduced Iba1 levels, which could suggest that Q134R directly affects microglial NFAT signaling. However, a similar reduction in Iba‐1 was observed in APP/PS1 mice when VIVIT expression was limited specifically to astrocytes using AAV vectors (Furman et al., 2012), highlighting the extensive cross‐talk between different cell types in the brain (and the difficulty of pinpointing cell‐type‐specific actions of pharmacologic compounds). Finally, NFAT signaling in other cell types including oligodendrocytes (Weider et al., 2018) and pericytes (Blanchard et al., 2020) has been recently proposed to modulate myelination and cerebrovascular pathologies found in progressive neurodegenerative diseases such as multiple sclerosis and AD. Together, these observations provide an extensive array of possible mechanisms through which Q134R may affect neural function.

### 3.4 Q134R and non‐NFAT targets

An earlier study found that Q134R‐stabilized HIF1A, a transcription factor induced during hypoxic conditions. Cerebral hypoperfusion and AD are bidirectionally linked suggesting that HIF‐1A may have a central role in AD pathophysiology (Ashok et al., 2017; Ogunshola & Antoniou, 2009). HIF1A‐dependent transcriptional regulation is well known to promote hypoxic adaptation, perhaps through an astrocyte‐based mechanism (Hirayama et al., 2015), which could provide compensatory neuroprotection during cerebral ischemia, and by corollary, in AD (Singh et al., 2012). Q134R‐mediated improvements in synapse function and cognition observed in this study may therefore arise from the stabilization of HIF1A and the promotion of HIF1A signaling. On the other hand, HIF1A has also been shown to drive AD pathophysiology through increased production, and/or reduced clearance, of Aβ peptides (Ashok et al., 2016; Pra et al., 2011; Zhang et al., 2007, Kim et al., 2017). HIF1A activation also transforms immunometabolism in glial cells, leading to the generation of pro‐inflammatory agents that promote glial reactivity and inhibit LTP (Yao et al., 2013; Arias‐Cavieres et al., 2020; York et al., 2021). Based on these observations, the promotion of HIF1A stability via Q134R would be expected to exacerbate microglial reactivity and LTP impairments in APP/PS1 mice, which is nearly the opposite of what we observed in this study. To complicate matters, it seems highly plausible that Q134R’s effects on neural function reflect a mixture of HIF1A activation and NFAT inhibitory effects. For instance, if HIF1A and NFAT4 both promote BACE1‐mediated Aβ production, as reported previously (Jin et al., 2012; Zhang et al., 2007), then the opposing actions of Q134R on these pathways may offset one another, resulting in little to no modulation of Aβ pathology (Figure 4e). There are also putative interactions between the HIF1A and NFAT transcriptional regulation (Siefert et al., 2009; Field et al., 2018) that may be modulated by Q134R resulting in complex biological outcomes. Clearly, additional studies will be needed to tease apart Q134R’s actions on HIF1 and NFAT‐signaling outcomes.

## 4 SUMMARY AND CONCLUSIONS

Q134R is a novel small chemical compound with cytoprotective and cognitive enhancing properties. The present work suggests that the beneficial effects of this compound may be related to inhibition of aberrant CN/NFAT signaling, which has been implicated by several research groups as a mechanism for AD pathophysiology. While identifying the precise mechanism of action for Q134R was beyond the scope of this study, future work is needed to clarify the actions of Q134R on multiple neural cell types and multiple NFAT isoforms. Nonetheless, the overall safety and efficacy of Q134R would seem to make this drug a prime candidate for further investigation in human clinical trials as a primary therapeutic, or at the least, as a compliment to other therapeutics, including other CN inhibitors.

## 5 MATERIALS AND METHODS

### 5.1 Q134R

Q134R was synthesized by Avidin Ltd. as described previously and used in cell culture conditions at similar concentrations (Hackler et al., 2019). The pharmacokinetic properties of Q134R were determined earlier during preclinical development using radioactive‐labeled Q134R (CLINICAL INVESTIGATOR'S BROCHURE, Q134R‐K, January 12, 2016). In male rats, organ/tissue concentrations of total radioactivity [μgE/g were measured (Dose: 10 mg/kg [14C]‐Q134R orally as potassium salt). The absorbed radioactivity showed relatively weak distribution properties. At 1‐hr post‐dose, tissue concentrations of Q134R (0.879 μgE/g) were markedly higher than the blood concentration. The brain levels of Q134R were similar to the blood level (0.963 μgE/g). The determined peak concentration in the brain was comparable to effective concentrations determined in vitro for NFAT inhibitory activity.

### 5.2 Primary astrocyte cultures and drug treatments

Primary cortical astrocytes were prepared from embryonic mouse pups and grown to confluence as previously described (Furman et al., 2010; Sama et al., 2008). Once cells were confluent, cultures were washed twice with Ca^2+^ and Mg^2+^‐free phosphate‐buffered saline and put into serum‐free medium overnight, approximately 16 hr. Cyclosporine A (CsA, Sigma‐Aldrich Corp. St. Louis, MO, USA) and Q134R (Avidin Ltd, Szeged, Hungary) were each prepared into DMSO stocks at 10 mM. CsA stocks were prepared fresh for each experiment and added to cells (at 1 µM) approximately 16 hr before treatment with varying concentrations of NFAT‐stimulatory agents: that is, phorbol ester/ionomycin, IL‐1β, or oAβ peptides. Ionomycin and phorbol ester (EMD Millipore) were each prepared as a 2 mM stock and used at a concentration of 1 μM. IL‐1β (Pepro Tech) was prepared as a 20 μg/ml stock solution in 0.1% bovine serum albumin (BAS) and used at a final concentration of 10 ng/ml. Synthetic Aβ peptides (rPeptide) were dissolved in DMSO (100 μg/ml) and added to sodium phosphate buffer (50 mM NaPi, 150 mM NaCl, pH 7.5) at a final concentration of 2 μg/ml and incubated at room temperature for 2 hr to allow seeding and formation of peptide oligomers. The oligomerization process was stopped with the addition of 1% BSA. One milliliter of preparation was loaded onto a G‐75 column and run in minimal essential medium (MEM, with 2 mg/ml BSA), and 1 ml fractions were collected and peptide concentrations determined by sandwich ELISA as described previously (LeVine, 2004). CN autoinhibitory peptide (AIP, from EMD Millipore) was prepared as a 500 μM stock in serum‐free cell culture medium and used at a final concentration of 20 μM. For cells treated with Q134R, a 10 mM stock (in DMSO, stored at −20℃) was thawed, and added to cultures at a final concentration of 10 µM one hour prior to treatment with NFAT stimulatory agents.

### 5.3 NFAT luciferase assays

Primary astrocytes were prepared as described above, except an NFAT luciferase adenovirus (Ad‐Luc) was added to the serum‐free medium so that the cells were infected with 50 MOI of virus per our previous studies (Furman et al., 2010; Sama et al., 2008). Inhibitors and activators were added as above, using the indicated concentrations of Q134R for dose‐inhibition curves. Cells were maintained in NFAT stimulatory agents for 4–5 hrs in serum‐free medium, then harvested for luciferase assays by washing cultures twice with Ca^2+^/Mg^2+^‐free phosphate‐buffered saline, then adding M‐PER Mammalian Protein Extraction Reagent (Thermo Scientific, Waltham, Massachusetts) to cells and immediately freezing overnight at −20℃. The next day, extracts were thawed and collected and used immediately in luciferase assays as per manufacturer's directions (ThermoFisher, Extended‐Glow Luciferase Reporter Gene Assay System, Catalog number T1033).

### 5.4 Western blot

Cultures were placed on an ice pack and washed twice with PBS with cell‐permeable phosphatase inhibitors (Activ Motif, Carlsbad, CA). After washing, 0.4 ml of phosphatase‐inhibitor buffer was added to each well and cells were scraped and collected into a 2 ml microfuge tube, pooling 3 x 35 mm cultures per tube. Cells were centrifuged, taken up in 156 µl of a sucrose buffer containing a panel of protease and phosphatase inhibitors: 0.25 M sucrose, 100 mM Tris, pH 7.4, 20 mM EGTA, 20 mM EDTA, protease inhibitor cocktail (Calbiochem Cat No. 524625), phosphatase inhibitor cocktail (Calbiochem Cat No 539134), and calpain inhibitor cocktail (Calbiochem Cat No. 208733). After resuspending each pellet, 60 µl of 4X sample buffer and 24 µl of 1 M DTT were added and the samples were heated at 65℃ for 20 min.

Samples were then resolved on 4–20% Criterion gels and transferred to PVDF membranes using standard techniques. Blots were washed with PBS, blocked with Odyssey blocking buffer (LI‐COR, Lincoln, NE), and put into primary antibodies in equal volumes of blocking buffer and PBS‐Tween overnight: 1:2000 rabbit Na^+^/K^+^ ATPase (Abcam, loading control, Cat. No. ab76020), 1:1000 rabbit anti‐ Cx43 (Cell Signaling, Cat. No. 3512S), 1:500 mouse anti‐dephosphorylated Cx43 (Invitrogen, Cat. No. 138300). Primary antibodies were detected with 800‐mouse and 680‐rabbit secondary fluorescent antibodies (LI‐COR, Lincoln, NE) on an Odyssey Scanner (LI‐COR, Lincoln, NE). Band intensities were determined using Image Studio 2.1 software (LI‐COR, Lincoln, NE). The level of total Cx43 and dephospho‐Cx43 (dpCx43) was normalized to Na^+^/K^+^ ATPase in the same sample. For each experiment, expression of the untreated sample was set at 100%, and others were calculated relative to it.

### 5.5 In Vitro calcineurin phosphatase inhibition assay

In vitro CN activity was assessed using a kit from Abcam (Cat. No. ab139461) according to the manufacturer's directions. Briefly, a standard curve of known PO_4_^3−^ concentrations was established according to phosphate standards provided in the kit. A timecourse of CN‐dependent dephosphorylation of a phosphopeptide substrate (provided by the kit) was first carried out to determine optimal lead times for inhibition studies. Once optimized, CN activity was quantified under the following conditions: No inhibitor, 20 µM CN autoinhibitory peptide (CN‐AIP), or Q134R (1 and 10 µM). The amount of PO_4_^3−^ released from the phosphopeptide was estimated using a standard curve.

### 5.6 Acute Q134R delivery to mice and behavioral testing

All behavioral experiments and corresponding drug treatments and procedures were conducted in accordance with animal experimentation and ethics guidelines of the European Union (2010/63/EU), reviewed and approved by the Regional Animal Health Authorities, Csongrad County, Hungary, and by the Joint Local Ethics and Animal Welfare Committee of Avidin Ltd. in possession of an ethical clearance XXIX./128/2013. All mice were housed under standard conditions (a 12‐hr light/dark cycle with lights on from 07:00–19:00 hr) and had free access to chow and water until behavioral testing.

#### 5.6.1 APP/PS1 mice and Q134R treatment

Twelve‐month‐old male APP/PS1 mice and their wild‐type C57BL/6 J littermates received 4 mg/kg Q134R or vehicle twice daily (at 8.00 am and 4.00 pm) for 7 days via oral gavage. On day 7, mice were tested on the Y maze. Q134R was dissolved in 10% nonionic detergent solution. The nonionic detergent solution was prepared by mixing 3x volume of polyethylene glycol (average molecular weight 200, Sigma Aldrich, Germany) and 1 volume of Kolliphor® HS 15 (BASF, Germany) with 36x volume of sterile distilled water. Each day mice were weighed and the administration of Q134R or vehicle was carried out using 22G oral gavage syringes (Kent Scientific, USA).

#### 5.6.2 Intracranial delivery of oligomeric Aβ peptides and Q134R treatment

Preparation of oligomeric Aβ peptides, drug treatment, and Y maze testing was performed at Synaging (Nancy, France) according to previously published protocols (Bouter et al., 2013; Youssef et al., 2008). At four days prior to Y maze testing, 3‐month‐old C57BL/6 J mice were deeply anesthetized and placed in a stereotaxic frame. Freshly prepared oAβ peptides (50 pmol in 1 μL; 0.1 M phosphate‐buffered saline (pH 7.4)) or vehicle (0.1 M phosphate‐buffered saline) were injected into the right ventricle with stereotaxic coordinates from the bregma (AP −0.22, L −1.0 and D 2.5 in mm). Injections were made using a 10‐μl Hamilton microsyringe fitted with a 26‐gauge needle. After injections, mice were brought out of anesthesia and returned to home cages until behavioral testing. Starting on the day of oAβ administration, mice were treated with vehicle or Q134R (4 mg/kg, 2× daily) as described above and then treated for four additional days until Y‐maze testing.

#### 5.6.3 Novel arm Y‐maze test of APP/PS1 mice

Testing occurred in a Y‐shaped maze with three transparent plastic arms (55 × 15 × 20 cm) at a 120° angle from each other. There were different signs (circle, rectangle, and square) on the top of each arm and different figures beside the walls and at the end of each arm. Three different arms were specified in advance: “Start arm,” “Familiar arm,” and “Baited arm.” Two arms from the three (Start and Baited) could be blocked with a nontransparent wall. Seven days prior to the experiment, all animals were familiarized with the room, staff, smells, and noises. Each mouse was treated with a test drug or its vehicle two hours prior to its Y‐maze run. Experiments consisted of two sessions. In the first, or habituation, session one arm (later Baited arm) was blocked with a nontransparent wall. One hour later, in the second or test session, the Baited arm was opened. In both sessions, each mouse was allowed to explore the maze for 4 min, and between habituation and test trials mice were allowed to relax in their home cage. Each mouse started from the end of the Start arm. Dwell times were recorded for Familiar and Baited arms to calculate the percentage of “Relative time spent” in the novel‐baited arm [%Relative time spent = (Time spent in baited arm / Total time spent in arms) × 100].

#### 5.6.4 Spontaneous alternation Y‐maze test of intracranial injected mice

Testing occurred in a Y‐shaped maze with three light‐gray, opaque plastic arms (35 × 7 × 15.5 cm) at a 120° angle from each other. Seven days prior to the experiment all animals were familiarized with the room, staff, smells, and noises. Each mouse was treated with a test drug or its vehicle 2 hours prior to its Y‐maze run. During the test, each mouse was allowed to explore the maze for 4 min and we accepted a run as successful above 10 or more alternations (total alternations). Each mouse was started from the center of the maze. The number of arm entries and the number of triads are recorded to calculate the percentage of an alternation. An entry was counted when all four limbs are within the arm [% Alternation = (Number of Alternations)/(Total number of arm entries −2) *100].

Prior to initial use and between each session, the Y‐maze in both experimental setups was thoroughly cleaned with 70% EtOH, followed by dH2O. By using a ceiling‐mounted CCD camera, all trials were recorded with Ethovision XT10 Software (Noldus, Netherlands).

### 5.7 Chronic Q134R delivery to mice, blood counts, and survival analyses

#### 5.7.1 Blood cell counts

Adult (6mos) male and female mice were gently restrained and 50 µL of blood was drawn from the facial vein and stored in potassium EDTA‐coated micro tubes. Blood was collected immediately before the initiation of vehicle or Q134R treatment (baseline) and then again at 1 and 3 months after twice‐daily delivery of vehicle or Q134R (4 mg/kg) via oral gavage, as described above. Cell counts for white blood cells, lymphocytes, monocytes, neutrophils, red blood cells, and platelets were determined by an Abaxis VetScan HM5 Hematology Analyzer (Allied Analytic LLC., Tampa, FL), according to the manufacturer's instructions.

#### 5.7.2 Survival analyses

Female C57BL/6J mice (*n* = 16/group) were purchased from Jackson Laboratory (AnimaLab Ltd. Vác, Hungary). Mice were housed at initial densities of four per cage with free access to sterilized VRF‐1 food (Akronom Ltd. Budapest, Hungary) and water. Mice were held in individually ventilated cages (IVC System, Suzhou Fengshi Laboratory Animal Equipment Co. Ltd. China). Starting at approximately 12 months of age, Q134R (4 mg/kg) or vehicle was delivered twice daily via oral gavage, as described above. Treatments were continued for each mouse until all‐cause mortality. GraphPad prism was used to calculate median and 90% percentile survival values, as well as Hazard ratios.

### 5.8 Chronic Q134R delivery to mice and AD biomarker measures

#### 5.8.1 Animals and Q134R administration

Six‐month‐old APP/PS1 mice and their wild‐type (C57BL/6 J) littermates were treated with vehicle or 4 mg/kg Q134R 2× daily for 3 months via oral gavage as described above. Mice were housed 3–4 per cage under standard light/dark (12 hr:12 hr) conditions at the University of Kentucky and provided free access to water and food pellets. All animals were treated in accordance with the University of Kentucky Institutional Animal Care and Use Committee and according to NIH guidelines. At the endpoint, brains were collected and fixed in 4% paraformaldehyde in phosphate buffer, pH 7.4, and then saturated with 30% sucrose buffer. Coronal sections at 40 μm thickness were prepared using a microtome (Leica) and kept at −20℃ in cryoprotectant solution containing 25% ethylene glycol and 25% glycerin in 0.05 M phosphate buffer.

#### 5.8.2 Immunofluorescence confocal imaging

Free floating brain sections were labeled to visualize Aβ, Iba‐1, GFAP, and NFAT4. Briefly, sections were first washed three times (1X PBS/0.1% Triton X) for 10’, room temperature (RT), on a shaker. The sections were then blocked for peroxidase using 3% hydrogen peroxide in methanol for 30’, RT, on a shaker. DAPI stain was then incubated with the sections at a concentration of 1:1000 for 10’, RT, covered from light. All subsequent steps were light‐sensitive and protected from direct light. The sections were again washed 3x 10’ RT on a shaker. Blocking buffer was applied to the sections (1xPBS/0.1%Triton X + 0.03 g/ml bovine serum albumin) for 30’, RT, on a shaker. Primary antibody was applied to each section: biotin anti‐Aβ_1‐42_ (1:200, Biolegend, catalog # 803007), Anti‐Iba1 (1:200, Wako, catalog # LAP0868); Anti‐GFAP (1:200, Life Technologies, catalog # 130300), anti‐NFAT4 (1:75, Santa Cruz, catalog # SC‐8405), diluted to proper concentrations in blocking buffer and incubated overnight at RT, on a rotator. The next day, the sections were washed, and secondary antibodies were applied: anti‐rabbit 488 (1:200, abcam, catalog # ab150077), streptavidin 594 (1:200, Vector, catalog # S32356), secondary antibody for Iba1, GFAP, and NFAT4, overnight at RT, on a rotator. For the NFAT4 sections, they were again washed and incubated in the Tyramide superboost system according to the manufacturer's protocol (Invitrogen TSA 488 catalog # B40922) for 10’ RT. The sections were washed one last time before mounting on slides and coverslipped using ProLong Diamond Antifade mountant (Invitrogen).

#### 5.8.3 Imaging and analysis

For quantifying Aβ_1‐42_ plaque load, Iba1, GFAP, and NFAT4, mounted sections were imaged and digitized using the Axio Scan Z.1 (Zeiss). Each slide was captured in its entirety at 20× magnification using the fluorescence LED. Images were then analyzed using HALO software (Indica Labs). Briefly, the background fluorescence was subtracted from each slide at a constant value. Then, each coronal section was traced and then the software was used to calculate the area that contained positive stain as a percentage of the entire area traced. Because of extensive labeling in blood vessels, NFAT4 analyses included only the NFAT4 signal co‐localized with GFAP‐positive astrocytes. For representative images, pictures were taken using a Nikon confocal Tie2 microscope and NIS software (Nikon).

### 5.9 Chronic Q134R delivery to mice and electrophysiology

Six‐month‐old wild‐type male and female C57BL/6J and APP/PS1 mice received vehicle or Q134R (4 mg/kg, 2x/day via oral gavage) for 3 months, as described above.

#### 5.9.1 Acute brain slice preparation

All techniques for preparing acute brain slices and assessing hippocampal synaptic function are nearly identical to our previous protocols (Furman et al., 2012; Mathis et al., 2011; Sompol et al., 2017). Briefly, after 3 months of vehicle/drug treatment, mice were euthanized via CO_2_ asphyxiation and brains were rapidly removed and briefly (1–2 min) stored in ice‐cold oxygenated (95% O_2_/5% CO_2_ mix), Ca^2+^‐free artificial cerebral spinal fluid (ACSF) prior to sectioning. ACSF consisted of (in mM): 124 NaCl, 2 KCl, 1.25 KH_2_PO_4_, 2 MgSO_4_, 26 NaHCO_3_, and 10 dextrose, pH 7.4. Coronal brain slices (400 µm) were prepared on a Vibratome (Leica Biosystems, Richmond, IL) in ice‐cold oxygenated Ca^2+^‐free ACSF and then transferred to a custom, humidified interface chamber where they were stored in warmed (32℃) oxygenated ACSF containing 2 mM CaCl_2_ until electrophysiologic analyses of hippocampal CA3‐CA1 synaptic function.

#### 5.9.2 Population synaptic strength measures

Slices were transferred to a Kerr Tissue Recording system (Kerr Scientific Instruments) and submerged in warmed (∼32℃) oxygenated aCSF containing 2 mM CaCl2 and 2 mM MgSO_4_. Schaffer collaterals were activated with a bipolar stainless steel electrode located in *stratum radiatum*. Stimulus intensity was controlled by a constant current stimulus isolation unit (World Precision Instruments), and stimulus timing was controlled by LabChart 8 software (ADInstruments; RRID: SCR_001620). Field potentials were recorded in CA1 *stratum radiatum* using a Ag/AgCl wire located ∼1–2 mm from the stimulating electrode. Field potentials were amplified 100X and digitized at 10 kHz using the Kerr Tissue Recording System amplifier and a 4/35 PowerLab analog‐to‐digital converter (ADInstruments). To assess basal synaptic strength, 100 μs stimulus pulses were given at 12 intensity levels (range, 25–500 μA) at a rate of 0.1 Hz. Five field potentials at each level were averaged, and measurements of fiber volley (FV) amplitude (in mV) and EPSP slope (mV/ms) were performed offline using the LabChart 8 software. Synaptic strength curves were constructed by plotting EPSP slope values against FV amplitudes for each stimulus level. To calculate paired‐pulse facilitation (PPF), the average EPSP slope was measured along the linear portion of the synaptic strength curve (at 50‐100uA stimulation). Then, the EPSP slope of S2 was divided by the EPSP slope of S1 and multiplied by 100. The EPSP slope at which a population spike (PS) appeared was reported as a population spike threshold. Following synaptic strength curves, stimulus intensity for each slice was adjusted to generate a field potential of approximately 1 mV. Electrical stimulation of CA3 Schaffer collaterals then commenced at a rate of 0.033 Hz to acquire a baseline (approximately 20 min). LTP was induced by applying two 100 Hz stimulation trains, 1‐s duration, each train separated by 10 s. Synaptic responses were then elicited for an additional 60 min. For time‐plot illustrations, EPSP slopes in each slice were normalized to their respective baseline. EPSP slopes during the last 10 min of the post‐100 Hz baseline were averaged in each group statistical analysis by GraphPad Prism software. Synaptic parameters were averaged across slices within each animal to get a single data point per animal. Thus, *n* for each experiment reflects the number of animals included in each treatment group.

#### 5.9.3 Statistics

All statistical analyses were performed using GraphPad Prism v7 (GraphPad Software, San Diego, CA). Student's *t* tests were used to evaluate differences between the two treatment groups. Comparison of two or more genotype/treatment groups was made using one‐way or two‐way analysis of variance (ANOVA), followed by Fisher's LSD post hoc tests. The Log‐rank test and Gehan–Breslow–Wilcoxon test were used to compare survival curves between vehicle and Q134R‐treated mice. Significance for all statistical comparisons was set at *p* ≤ 0.05.

## 6 SUPPLEMENTARY METHODS

### 6.1 NFAT luciferase assays

#### 6.1.1 HEK 293 cells

HEK293 cells stably expressing an NFAT response element luciferase construct, were maintained in DMEM (Gibco) culture media containing 10% FBS (Gibco) at 37℃ in a 5% CO_2_ atmosphere. For the inhibition experiments, 2 × 10^4^ cells were seeded in 96 well plates and incubated overnight. Cells were treated with an increasing concentration of test compounds and incubated for 1 hr followed by activation with 0.5 µM Calcium Ionophore A23187(Sigma) and 0.5 µg/ml PMA (Phorbol myristate acetate, Sigma). After 5 hr, cells were lysed and luciferase activity was measured (Bright‐Glo™ Luciferase Assay, Promega). Cyclosporin A (Sigma) was used as a positive control. The results are presented as RLU (luminescence) corresponding to NFAT activity.

#### 6.1.2 Neurons

Primary cortical neurons were prepared as described previously (Sama et al., 2008). Briefly, cortical tissue dissected from E18 Sprague–Dawley rat pups was passed through a glass pipette and filtered through a 0.22‐micron nylon mesh. Following centrifugation, the pelleted cells were plated in neurobasal medium supplemented with B27, penicillin/streptomycin/neomycin, and glutamax onto poly‐lysine coated wells of a 6‐well plate at 150,000 cells per well. After growth for 7 days in a 5% CO2 incubator, cells were used in assays. Preloading with Ad‐NFAT‐luc and pretreatments with Cyclosporin A and Q1 were done as described above for astrocytes, and cells were treated with 20 mM KCl to depolarize neurons. After 4–5 hr in KCl, cells were harvested in M‐Per and analyzed in luciferase assays as described above for astrocytes. Statistical analyses were performed as described in Figure 1.

### 6.2 Immunofluorescence labeling for NFAT3 and MAP2B

APP/PS1 mice and WT littermates treated chronically with vehicle (WT vehicle, *n* = 12; APP/PS1 vehicle, *n* = 17) or Q134R (4 mg/kg, 2×/day P.O. for 3 months) (WT Q134R, *n* = 14; APP/PS1 Q134R, *n* = 20). Mice were 6‐months of age when treatment began. Brains were harvested and prepared for immunofluorescent labeling as described in Figures 4 and 5. Images were taken using a Nikon confocal Tie2 microscope and NIS software (Nikon). For each animal, an image of the CA1 region of the hippocampus was taken at 60x magnification, using the large image setting to take 2x2 image fields with a 5% overlap stitching. Furthermore, each image consisted of z‐stacks to encompass a 16 μm thickness for each section at 1 μm intervals (17 z levels total). After capturing the images, the amount of fluorescence was quantified for each label (DAPI, NFAT3, and MAP2b). Primary antibodies included: anti‐NFAT3 (1:2000) from Abcam (catalog # Ab99431) and anti‐Map2b (1:8000) from EMD Milliipore (catalog # mAB3418). Briefly, three 50 × 50 μm regions of interest were drawn over each image. Layers were created to mask the positive signal of each fluorophore within the region of interest and applied to each z‐level of the captured images. By combining the binary (fluorophore positive) areas across each z level, we obtained a volume of positive fluorophore signals for each region of interest. The three ROIs for each cellular region were then averaged together and reported. Statistical analyses were performed as described in Figures 4, 5.

## CONFLICTS OF INTEREST

L.G.P. is CEO of Avidin Ltd and the main stakeholder of Avidin Ltd and Aperus Pharma co. Ltd.; O.H. and L.H. are employees at Avidin Ltd. The remaining authors have no conflicts to report.

## AUTHOR CONTRIBUTIONS

PS, JLG, SDK, IAA, RC, EAC, SAK, GKN, JFA, OH, LIN, and LH participated in data collection. PS, JLG, SDK, LH, LGP, and CMN participated in data analyses. LGP provided Q134R compound for experiments. PS, JLG, LH, LGP, and CMN conceived of the study and wrote the manuscript.

## Supporting information

Fig S1

Fig S2

Fig S3

## Data Availability

The data that support the findings of this study are available from the corresponding author upon reasonable request.
