# Supplementary figures and images for: Q134R: Small chemical compound with NFAT inhibitory properties improves behavioral performance and synapse function in mouse models of amyloid pathology
Source: Aging Cell. 2021 Jun 12;20(7):e13416. doi: 10.1111/acel.13416 (PMC8282246; doi:10.1111/acel.13416)

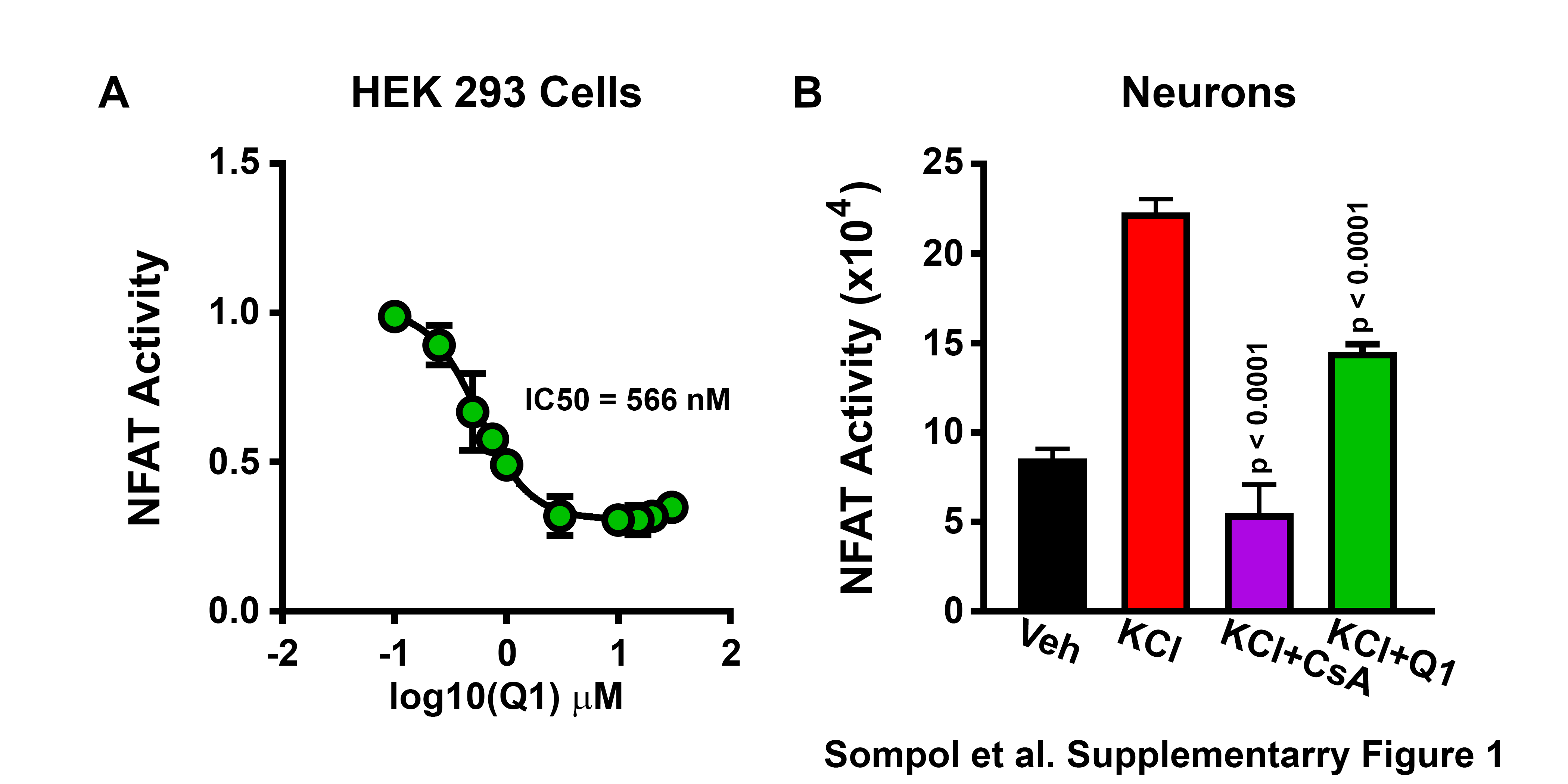

Supplement: Supplementary file 1 — Fig S1 [file ACEL-20-e13416-s001.tif]

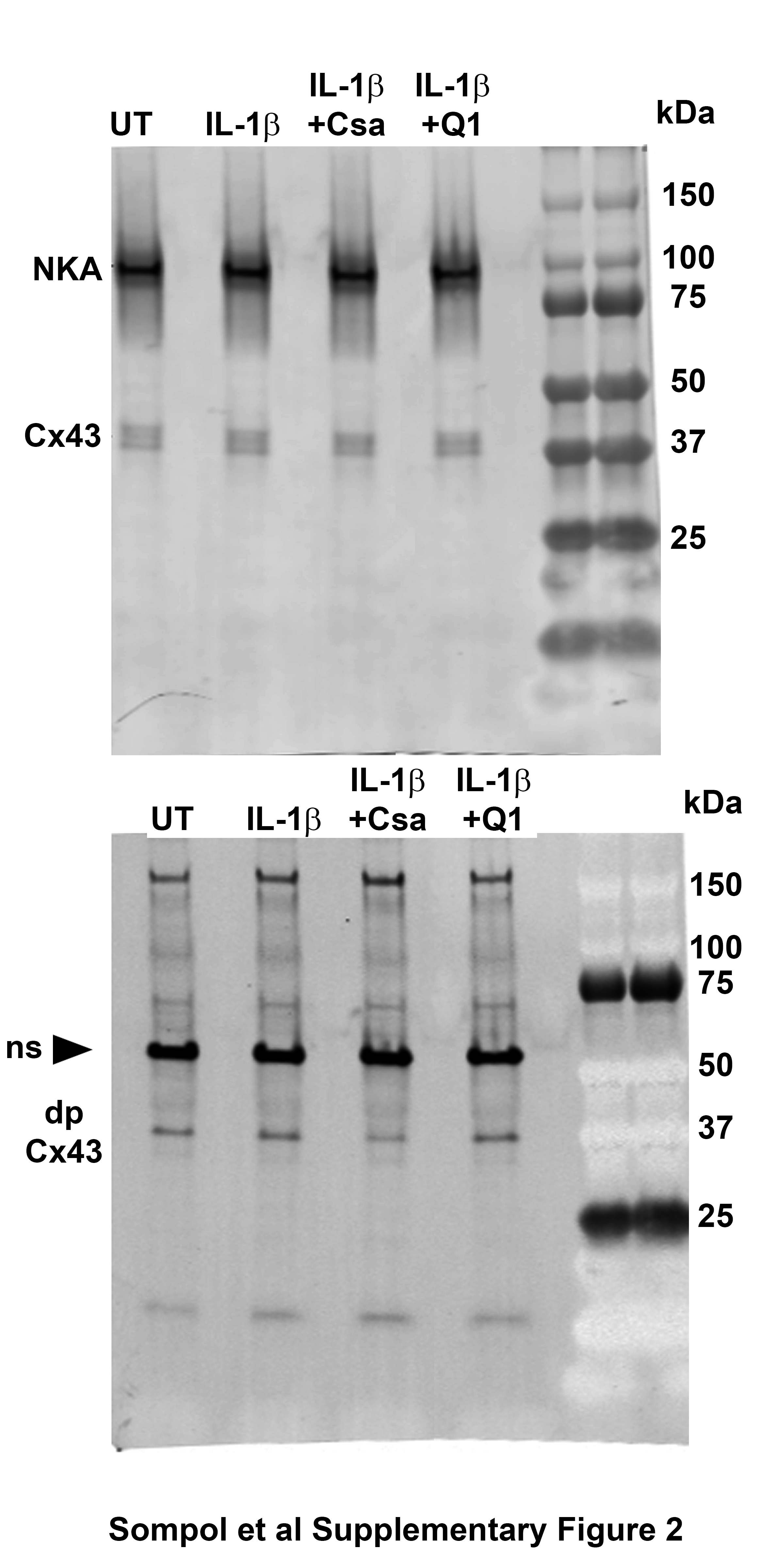

Supplement: Supplementary file 2 — Fig S2 [file ACEL-20-e13416-s002.tif]

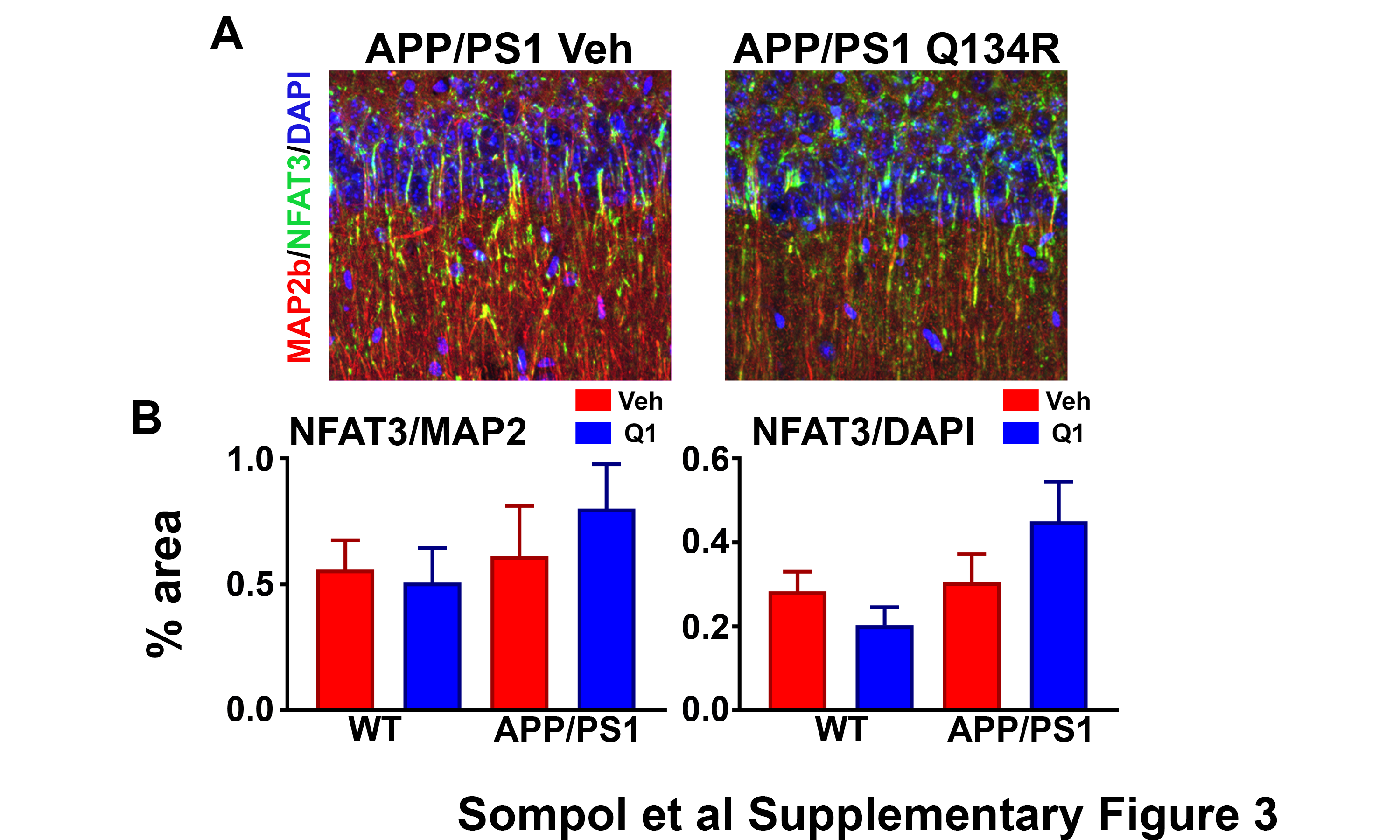

Supplement: Supplementary file 3 — Fig S3 [file ACEL-20-e13416-s003.tif]
